# Potentials of Antitussive Traditional Persian Functional Foods for COVID-19 Therapy^†^

**DOI:** 10.3389/fphar.2021.624006

**Published:** 2021-07-16

**Authors:** Ghazaleh Mosleh, Parmis Badr, Meysam Zaeri, Abdolali Mohagheghzadeh

**Affiliations:** ^1^ Phytopharmaceutical Technology and Traditional Medicine Incubator, Shiraz University of Medical Sciences, Shiraz, Iran; ^2^ Pharmaceutical Sciences Research Center, Shiraz University of Medical Sciences, Shiraz, Iran; ^3^ Department of Phytopharmaceuticals (Traditional Pharmacy), School of Pharmacy, Shiraz University of Medical Sciences, Shiraz, Iran

**Keywords:** antitussive, functional foods, traditional Persian medicine, phytochemical, COVID-19

## Abstract

Coronavirus disease 2019 is a worldwide pandemic resulting in a severe acute respiratory syndrome. Remdesivir is the only FDA-approved drug for hospitalized patients older than age 12. It shows the necessity of finding new therapeutic strategies. Functional foods (FFs) could have co-therapeutic and protective effects against COVID-19 infection. Traditional Persian medicine (TPM), one of the safest and most popular schools of medicine for hundreds of years, has recommended potential FF candidates to manage such a global pandemic. To reveal the potential of TPM in terms of antitussive FFs, traditional Persian pharmacopoeia “Qarabadin-e-Salehi” was searched using the keywords “*Soaal*” and “*Sorfeh*.” Also, a search of MEDLINE, PubMed Central, Google Scholar, and Science Direct was performed for the relevant literature published from the inception up to March 2021. A combination of search terms including “cough, antitussive, antioxidant, anti-inflammation, antiviral, COVID-19, mucoactive, mucolytic, expectorant, and mucoregulatory” was also applied. The potential mechanism of action in SARS-CoV-2 infection was discussed. Twelve TPM FFs were found including Laooqs, Morabbas, a Saviq, a soup, and a syrup. They are combinations of two to seven ingredients. Natural compounds of mentioned formulations have the main pharmacological mechanisms including antiviral, anti-inflammatory, antioxidant, antihistamine, bronchodilator, immunomodulatory, and mucoactive effects as well as central or peripheral antitussive activities. FFs are cost-effective, easily accessible, and safe options for both treatment and prevention of COVID-19. They might have positive psychological effects along with their pharmacological effects and nutritional virtues. They could also manage persistent respiratory discomforts after recovery from COVID-19.

## Introduction

Coronavirus disease 2019 (COVID-19) is a worldwide pandemic resulting in a sudden increase in hospitalizations due to pneumonia and damages to different organs (Wiersinga et al., 2020). This viral pulmonary infection occurs when respiratory mucosa cells are infected by the SARS-CoV-2 virus. The epithelium of the respiratory tract is composed of various cells including goblet cells producing mucus as the first barrier of the incoming viruses, ciliated cells, non-ciliated cells, and club cells producing proteases. After the attachment of S spike protein of the virus to the ACE2 receptor of host cells, the virus penetrates into the cells (Azer, 2020; Subbarao and Mahanty, 2020; Wiersinga et al., 2020). Then, pro-inflammatory cytokines and type I interferons are expressed to induce an antiviral state. The cytokine storm condition is seen after immune response dysregulation in severe SARS-CoV-2 cases (Subbarao and Mahanty, 2020). Also, lung edema and necrotic changes can be seen as the results of proinflammatory cytokines (Azer, 2020). Most of the infected patients get a mild to moderate pulmonary illness, whereas others experience severe pneumonia. Common mild COVID-19 symptoms are sore throat, cough (usually dry cough, but in some cases phlegmatic cough), headache, fever, myalgia, fatigue, anosmia, anorexia, and diarrhea. Severe COVID-19 is associated with pneumonia, dyspnea, confusion, pain in chest, fever, and anorexia (Clemency et al., 2020; Donma and Donma, 2020; Struyf et al., 2020). The most common symptom of COVID-19 is dry cough (Li and Ma, 2020). As coughing is one of the main modes of viral transmission among individuals, it is supposed that viruses have developed cough mechanisms to enhance their transmission. For instance, a virus can induce coughing by selective changes in neural signaling. In addition, stimulated mucus production by a virus can induce coughing, at least by irritation sense in the airway tract (Footitt and Johnston, 2009). Epidemiologic studies reveal that droplet expulsion during coughing is one of the most common routes of COVID-19 transmission (Wiersinga et al., 2020). In fact, although cough is a defensive reflex of lungs to clear the respiratory system, excessive cough can trigger a wide range of complications including respiratory, cardiovascular, GI, neurologic, constitutional, genitourinary, musculoskeletal, ophthalmologic, dermatological, and psychosocial problems (Nosalova et al., 2006; Irwin et al., 2020). Several adverse events such as headache, laryngeal trauma, pulmonary interstitial emphysema, and brady- or tachyarrhythmias are reported as possible complications induced by excessive cough in COVID-19 patients (Jalali et al., 2020). Also, throat pain, throat scratching, and difficulty in swallowing are common complications associated with coughing (Chiru et al., 2020). Dry cough is a common symptom prevalent in 60–86% of hospitalized cases (Carfì et al., 2020). Persistent cough could decrease life quality by interfering with normal activities and sleep (Weinberger and Lockshin, 2017). Chronic cough could occur in months after recovery from COVID-19, and it may lead to substantial community morbidity (Fraser, 2020). Hence, this concerning situation would require an optimal management for future public health. Many antiviral drugs including favipiravir, lopinavir-ritonavir, ribavirin, and hydroxychloroquine have shown poor efficacy in the treatment of COVID-19 (Martinez, 2020). In October 2020, remdesivir (the potent antiviral agent inhibiting RNA-dependent RNA polymerase) received FDA approval for hospitalized patients older than age 12 (Gordon et al., 2020; U.S. Food and Drug Administration, 2020). Besides, several COVID-19 vaccines are developed and currently evaluated in human trials (Lazarus et al., 2021). Although discovering a safe and effective vaccine is the best solution to manage coronavirus disease 2019 (Matteo et al., 2020), other therapeutic strategies such as using traditional medicine prescriptions could be a solution for local people, before a safe and effective vaccine or/and drug is available.

Functional foods (FFs) have possible co-therapeutic and protective effects against the COVID-19 virus (Matteo et al., 2020). These food and drink traditional formulations are natural that are taken as part of one's daily diet yielding physiological benefits that may help to enhance body health and well-being (Roberfroid, 2002; Krystallis et al., 2008). Since many people of the world are confined to their homes in these quarantine days, inclusion of available natural foods in their daily diet could be a rational suggestion to enhance the immunity of their body against COVID-19. This might decrease the risk of the infection in healthy people and also increase the rapid recovery of patients after SARS-CoV-2 infection (Yang et al., 2020).

Traditional Persian medicine (TPM) is a famous medical doctrine based on humors which are special bodily fluids required for the physiological functioning of each organism (Sirasi, 1990; Hamedi et al., 2013). General health status in TPM is regulated by the equilibrium of four humors including blood, phlegm, bile, and black bile (Alam et al., 2020). According to TPM, diet is a very important factor because food can be converted into the bodily humors. Each food has its particular qualities, and its excessive consumption can induce extreme quantities of one special humor (Jackson, 2001). Food intake for medical purposes has a long history in TPM deliberating foods essential not only for energy providing, but also as a factor to affect the humoral balance of the body. In medieval Persia, the great physicians such as Rhazes (854–921 A.D.) and Avicenna (980–1037 A.D.) wrote the first manuscripts about diet, nutrition, and health regimes. They considered nutrition as an independent and highly developed medical science (Nikaein et al., 2012). In fact, TPM has a rich cuisine presenting diverse recipes for different kinds of FFs (Gharibzahedi, 2018). According to the TPM point of view, if a disease can be treated with food, medicine should not be administered. Furthermore, there are many FFs in TPM which are recommended to accompany the medications (Amiri Ardekani et al., 2020). Actually, TPM has categorized foods, drugs, and their intermediate formations into five general groups including *Ghaza-e-Motlaq* (absolute aliment), *Ghaza-e-Davai* (FF), *Dave-e-Ghazai* (pharmaconutrient), *Dava-e-Motlaq* (absolute medicament or drug), and *Sam-e-Motlaq* (poison). This classification is comparable to that of modern medicine (i.e., nutrients, FFs, nutraceuticals, and poisons) (Soleymani and Zargaran, 2018). Indeed, medicine and food are shaded into each other as recorded by TPM. Avicenna asserted a distinction between food and medicine, indicating that food is a substance assimilated by the body, while medicine assimilates the body to itself. But both medicine and food can affect the body of the person who consumed them (Sirasi, 1990).

TPM Qarabadinic manuscripts are traditional pharmacopoeias containing many multi-ingredient formulations some of which are FFs. For instance, Qarabadin-e-Salehi (Amale Saleh) written by Mohammad Saleh Ghaeni Heravi in 1766 A.D. is a complete and comprehensive Persian language pharmacopoeia on TPM formulations. It could be defined as an example of Persian literature which is written prior to the replacement of TPM by Western medicine in Iran and it has a unique place among traditional pharmacy manuscripts (Zarshenas et al., 2013; Badr et al., 2014; Farjadmand et al., 2017). TPM has recommended numerous natural formulations to manage such respiratory discomforts. TPM antitussive formulations are categorized into two major classes. One could modify the major cause (*Ezaleh-sabab*) of disease such as infective humors or local inflammations and the other could relieve cough symptomatically. In addition, TPM believes that using antitussive agents is necessary when cough occurs during fever, if not, it may result in persistent fever in patients (Avicenna, 1025). So, antitussive formulations mentioned in TPM manuscripts are recommended for both prevention and treatment of cough and its relevant discomforts. In this regard, the present study introduces traditional Persian antitussive FFs with a review on their potential healing effects against COVID-19 through the recent evidence-based published articles.

## Methods

In this study, the research was done in two steps presented as follows:

A. The potential antitussive FFs recommended in TPM.
B. Efficacy and pharmacological mechanisms related to FF ingredients for antitussive properties in COVID-19.

### Section A

In the first step, traditional FFs for the treatment or prevention of dry cough recommended in TPM were introduced. For this purpose, the literature in Qarabadin-e-Salehi, one of the most complete and recent books on TPM compound remedies, was searched using the keywords of cough (“*Soaal*” and “*Sorfeh*” in Persian). Twelve recommended FFs were found and the traditional names of plants were matched with the current scientific plant names using a book providing the scientific names of TPM plants in accordance with their morphological descriptions (Ghahraman and Okhovvat, 2004). In the next step, scientific names were validated according to The Plant List website (The Plant List, 2013). The traditional temperaments of natural ingredients were defined according to “Makhzan-al-Adviah” book (Aghili Khorasani et al., 1771).

### Section B

Pharmacological studies related to 22 natural ingredients of the selected FFs were gathered through search of MEDLINE, PubMed Central, Google Scholar, and Science Direct by the combination of the scientific names or common names of each ingredient with “cough, antitussive, antioxidant, anti-inflammation, antiviral, COVID-19, mucoactive, mucolytic, expectorant, and mucoregulatory.” Also, the relevant studies about the isolated chemical compounds of each ingredient were included. All data gathering and literature research were done from the inception until March 2021. Articles published in English were only considered.

## Results

TPM has described particular FFs for respiratory disorders. In this article, five types of antitussive Persian FFs including Laooqs, Morabbas, Savighs, soups, and syrups have been discussed. These FFs have different textures and processing methods. The antitussive mechanisms of the mentioned TPM FFs in this article could be summarized to the following four aspects. ⅰ) Mucoactive functions by mucolytic properties (stimulation of ciliary beating, decreasing the viscosity of mucus, and viral adhesiveness), expectorant effects (increasing mucus secretion, gastro-pulmonary reflex, hydration of the airway mucus), and mucoregulatory activities (normalizing mucus, emptying mucus glands, and enhancing ciliary transport). ⅱ) Central antitussives effects through brainstem sensory afferents. ⅲ) Peripheral antitussive properties through C-fiber sensory afferents. ⅳ) Other antitussive mechanisms including anti-inflammatory, antioxidant, antiviral, antihistamine, bronchodilator, and immunomodulatory effects (Footitt and Johnston, 2009; Zanasi et al., 2020). More details about the mentioned TPM antitussive FFs listed alphabetically are described as follows:

### Laooq

Laooq, a semisolid traditional formulation, includes powdered medicinal plants in honey or a viscous syrup. Being similar to linctus, Laooq can be considered a dosage form specifically prescribed for the respiratory system. It has been used orally through licking, and its high viscosity leads to longer transit time through esophagus. The ingredients are often demulcents and antitussive agents (Zarshenas et al., 2013).

#### Garlic Laooq

**FIGURE 1:**
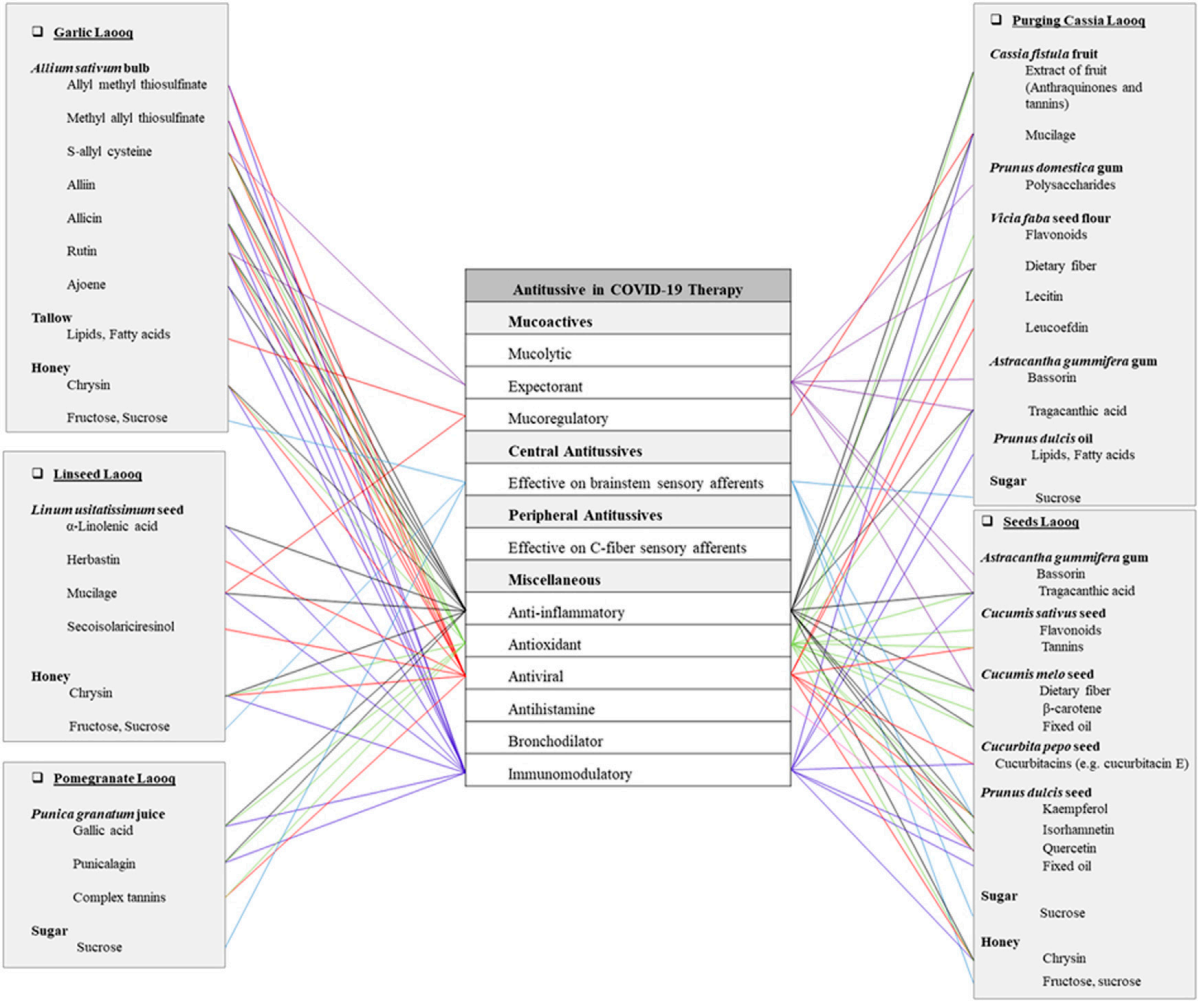
Potentials of antitussive Persian Laooqs in COVID-19 therapy.

Garlic Laooq is made of *Allium sativum* L. (Amaryllidaceae) cooked bulb (hot and dry temperament), tallow (hot and dry temperament), and honey (hot and dry temperament). It has been traditionally recommended for removing phlegm from lungs (Ghaeni Heravi, 1766). Garlic contains enzymes comprising allinase, peroxidases, and myrosinase. Also, garlic has sulfur-containing compounds including alliin, allicin, allylpropyl disulfide, diallyl disulfide, diallyl trisulfide, ajoene, and vinyldithiines. Its terpenes are α- and β-phellandrene, citral, geraniol, and linalool. Other constituents of garlic include proteins such as glutamyl peptides, amino acids such as arginine and glutamic acid, volatile oils, minerals, lipids, prostaglandins (A2, D2, E2, F1a, F2) trace elements, and vitamins (Barnes et al., 2013). One of the main indications of garlic has been for pulmonary diseases and coughs (Papu et al., 2014). Garlic has shown antiviral properties, specifically against influenza virus, parainfluenza virus type 3, herpes simplex viruses, vaccinia virus, and vesicular stomatitis virus. Its main virocide constituents are ajoene, allicin, allyl methyl thiosulfinate, and methyl allyl thiosulfinate. Garlic supplements have prevented common cold viruses (Singh and Singh, 2008). Its phytochemicals have anti-inflammatory and antioxidant activities. They inhibit the production of free radicals, increase cellular antioxidant enzymes, support endogenous radical scavenging activities, and suppress the activity of NF-kB. *In vitro*, the extract of aged garlic and s-allylcysteine blocked the oxidation of low-density lipoprotein and it could protect endothelial cells of pulmonary artery against injury induced by oxidized low-density lipoprotein (Barnes et al., 2013). Also, s-allylcysteine increases the mucus secretion (Park et al., 2014). *In vitro* and *in vivo* studies have shown that garlic has several immune-boosting effects including macrophage phagocytosis induction and lymphocyte proliferation, stimulation of lymphocyte- and macrophage-infiltration into transplanted tumors, induction of the release of interferon-γ, and increase in natural killer cell activity and interleukin-2 production. Ajoene has shown *in vitro* inhibitory effects on the release of lipopolysaccharide-induced PGE2 in macrophages due to the inhibition of COX-2 activity (Barnes et al., 2013). Also, it is reported that the production of COX-2 and PGE2 is prevented by NF-κB inactivation (El-Saber Batiha et al., 2020). Moreover, emigration of neutrophilic granulocytes into epithelia is inhibited by garlic extracts (El-Saber Batiha et al., 2020). Garlic phytochemicals such as S-allyl cysteine, alliin, and allicin have shown antiviral, antifibrotic, antioxidant, anti-inflammatory, and immunomodulatory properties in recent studies. Allicin has shown dual S-thioallylation of SARS-CoV-2 M pro in a recent *In silico* study (Shekh et al., 2020). Garlic stimulates natural killer cell activity and keeps the immune homeostasis by its sulfur-containing compounds. Furthermore, allilin has shown the positive effects to prevent intra-alveolar edema and decrease inflammatory cytokines as well as neutrophils infiltration into the alveolar region. Additionally, sucrose methyl 3-formyl-4-methylpentanoate, another phytochemical in garlic, has shown inhibitory effects on the alveolar damage, lung infection, and thrombotic lesions. Generally, these preclinical studies demonstrate the efficacy of garlic in respiratory infections, alveolar edema, sepsis, pulmonary fibrosis, and acute lung injury, all of which are common symptoms in advanced COVID-19 patients (Thota et al., 2020; Oladele et al., 2020). In addition, garlic enhances the immune system through the reduction of leptin levels which has proinflammatory characteristic (Donma and Donma, 2020). Rutin has shown stimulatory effects on mucus secretion in recent studies (Jeong, 2009). Rutin isolated from garlic has a binding affinity toward COVID-19 main protease (Majumder and Mandal, 2020). Furthermore, a molecular docking analysis highlighted that garlic organosulfur essential oil components could have strong interactions with the main protease PDB6LU7 of coronavirus 2 and ACE2 amino acids. These findings reveal that garlic could contribute to block the coronavirus invasion in the body (Figure 1) (Thuy et al., 2020). The other ingredient of garlic Laooq is tallow. Chemical components of tallow are lipids and fatty acids comprising oleic, palmitic, stearic, myristic, and linoleic acids. These constituents are responsible for emollient properties (Leung, 2015; Kelm and Wickett, 2017).

#### Linseed Laooq

Linseed laooq is made of *Linum usitatissimum* L. (Linaceae) seed (hot and dry temperament) and honey (hot and dry temperament). It has been traditionally recommended for dry cough (Ghaeni Heravi, 1766). Linseed contains cyanogenic glycosides comprising diglucosides linustatin, neolinustatin, and some linseed samples have linamarine in trace amounts. Fixed oil in linseed is identified as α-linolenic acid (45–50%), linoleic acid (16–20%), oleic acid (18–24%), palmitic acid (5–7%), and stearic acid (0.25–5%). Furthermore, minor constituents of flavonoids such as herbacetin and kaempferol derivatives have been detected in linseed. Besides, polysaccharides including arabinoxylane and rhamnogalacturonan, and other constituents for example nonprotein aminoacids, protein, sterols, tocopherols, and several phenolic compounds such as p-coumaric and caffeic acids have been reported in linseed (Barnes et al., 2013). Linseed has various types of lignans such as (+)-pinoresinol, (+)-lariciresinol, and (−)-matairesinol, as well as secoisolariciresinol diglucoside that has shown antiviral properties in recent studies (Barnes et al., 2013; Chhillar et al., 2020). The seeds contain both mucilage and oil with laxative effects (Uden et al., 1994). Mucilage is a large hydrophilic polysaccharide with a highly branched structure that is able to trap water to form a gel in bronchial mucosa. The muco-adhesive properties of these polysaccharides are responsible for treating cough indirectly by the modulation of the sensitivity of cough receptors to protect them from local irritations as well as their local soothing actions and demulcent properties (Nosalova et al., 2013). Mucilage has immunomodulatory and anti-inflammatory effects (Bokov et al., 2020). Indeed, mucilage can prepare polysaccharide layers on inflamed epithelial tissue to protect and rehydrate it. In a recent study, oral administration of rhamnogalacturonan has shown significant effects in reduction of cough frequency and intensity (Mahboubi, 2020). In one *in vivo* study, rhamnogalacturonan promoted the expectoration and reduced the intensity and frequency of cough attacks (Nosál'ova et al., 1992). Also, arabinoxylan polysaccharide in linseed stimulates the immune responses. The pectin of linseed extracted in the acidic fraction of seed has a rhamnogalacturonan basis with lubricating properties suitable for using in demulcent and emollient substance (van Dam et al., 2017). Linseed tea is a suitable drink to relieve cough, cold symptoms, and bronchitis (Uden et al., 1994). Plants rich in polysaccharides such as *L. usitatissimum* show antioxidant properties (Kardošová and Machová, 2006; Kaithwas and Majumdar, 2012). Linseed oil has shown inhibitory effects on leukotriene-, histamine-, PGE2-, and bradykinin-induced inflammation. It blocks local vasodilatation, capillary permeability, leucocytes migration, and exudation during inflammation. Also, linseed decreases the expression of COX-1 and COX-2 significantly (Akbar, 2020). The results from an *in vivo* study have shown immunomodulatory effect of α-linolenic acid that increased INF-γ, and the ratio of INF-γ/IL4 as well as index of Th1/Th2 decreased IL-4, and preventive effect on tracheal responsiveness and inflammatory markers comparable to dexamethasone (Kaveh et al., 2019). In a FRET-based screening method, it was suggested that herbacetin (3,4′,5,7,8-pentahydroxyflavone) isolated from linseed may have proteolytic activity when tested against 3CL protease of coronavirus 2 (Figure 1) (Solnier and Fladerer, 2020).

#### Pomegranate Laooq

This Laooq is made of *Punica granatum* L. (Lythraceae) concentrated juice (cold and wet temperament) and sugar (hot and dry temperament). It has been traditionally recommended for coughs associated with hot distemprament (Ghaeni Heravi, 1766). Pomegranate has anthocyanins comprising delphinidin 3,5-diglucoside, cyanidin 3,5-diglucoside, pelargonidin 3,5-diglucoside, delphinidin 3-glucoside, a cyanidin–pentoside–hexoside, cyanidin 3-glucoside, cyanidin 3-rutinoside, pelargonidin 3-glucoside, and a cyanidin-pentoside. Its gallotannins include monogalloyl-hexoside and digalloylhexoside. Its ellagitannins include ellagic acid and its derivatives, galloyl-HHDP-hexoside, pedunculagin I and II, casuaricitin, valoneic acid, pomegranate gallagic acid, and gallagyl esters include punicalin and punicalgin. Hydroxybenzoic acids of pomegranate include gallic acid and protocatechuic acid, while its hydroxycinnamic acids contain caffeoyl hexoside, chlorogenic acid, *p*-coumaric acid, and their derivatives. Also, a dihydroflavonol named dihydrokaempferol-hexoside has been isolated from pomegranate juice (Fischer et al., 2011). Pomegranate phytochemicals have shown positive effects in the management of pulmonary inflammation (Shekhar et al., 2017). Pomegranate has shown inhibitory effects on inflammatory pathways such as NF-k B pathway. Indeed, pomegranate juice is rich in polyphenols having high antioxidant and anti-inflammatory properties *in vitro* and *in vivo*. Pomegranate juice has various effects such as increasing serum antioxidant capacity, reducing inflammation, and decreasing the activity of angiotensin-converting enzyme. According to a recent study, the antioxidant activity of total pomegranate juice was superior to its purified polyphenols. Therefore, it represents the chemical synergy and multifactorial effects of pomegranate whole extract compared to its single active ingredients. Gallic acid and punicalagin in pomegranate juice stimulate the expression of macrophage PON2 and the activation of PAPR gamma and AP-1 transcription factors. Daily consumption of pomegranate juice increases the antioxidant and antimicrobial capacities in the immune system (Reddy et al., 2007; Charles, 2013). Punicalagin, which is a polyphenolic compound isolated from pomegranate, has shown considerable positive *in vivo* effects to inhibit lung edema, inflammatory cell infiltration, and pro-inflammatory cytokines (IL-6, TNF-α, and IL-1β) discharge (He et al., 2020). Pomegranate has numerous applications for asthma, bronchitis, fever, cough, and inflammation (Reddy et al., 2007; Lansky and Newman, 2007). *Punica granatum* is used as a main component in local medications against cold, cough, and fever (Ballabh and Chaurasia, 2007). Pomegranate fruit is a rich source of antioxidants (Syed et al., 2013). On the other hand, pomegranate peel extract could inhibit myeloperoxidase production to reduce lungs inflammation. Another study on pomegranate fractions revealed reduction of neutrophils recruitment in the lung area and inhibition of changes in vascular pulmonary permeability. Furthermore, tannins available in pomegranate possess antioxidant and antimicrobial secretion activities (Figure 1) (Reddy et al., 2007; Shekhar et al., 2017).

#### Purging Cassia Laooq

This Laooq is made of *Cassia fistula* L. (Leguminosae) fruit (hot and wet temperament), *Prunus domestica* L. (Rosaceae) bark gum (hot and dry temperament), *Vicia faba* L. (Leguminosae) seed flour (cold and dry temperament), *Astracantha gummifera* (Labill.) Podlech (Leguminosae) gum (moderate and wet temperament), and *Prunus dulcis* (Mill.) D.A.Webb (Rosaceae) seed oil (moderate and wet temperament). It has been traditionally recommended for cough and pulmonary infections (Ghaeni Heravi, 1766). Methanol extract of *C. fistula* fruit pulp consists of flavonoids, saponins, steroids, triterpenoids, glycosides, anthraquinones, tannins, gums, amino acids, and mucilage. This extract demonstrated significant antioxidant activity. Pulp also contains antifungal constituents, betulinic acid, b-sitosterol, stigmasterol, ergosterol, fucosterol, lupeol, α-amyrin, and friedelin (Akbar, 2020). According to recent studies, *C. fistula* has laxative, antimicrobial, antioxidant, anti-inflammatory, and anti-pyretic properties. Also, it can control nasal infections and coughs (Tanveer et al., 2019; Pawar and Killedar, 2017; Nikhat and Fazil, 2020). Methanol extract of *Cassia fistula* showed significant antitussive activity (Bhakta et al., 1998). Stigmasterol has shown suppressing effects on allergen-induced asthma (Antwi et al., 2017). On the other hand, *Prunus dulcis* gum is a mixture of high-molecular polysaccharides such as hemicelluloses compounds having antitussive properties (Bouaziz et al., 2017; Bouaziz et al., 2015). *Vicia faba* L. is another ingredient of purging cassia Laooq. Seeds of *V. faba* are rich in proteins (globulins, albumins, and glutelins), carbohydrates, vitamins, folic acid, niacin, dietary fiber, and macro and micro nutrients. According to a recent report, dietary fiber has potential beneficial effects on lungs such as reducing inflammation and enhancing the antioxidant processes. It has been suggested that a high-fiber diet might reduce the occurrence of chronic cough symptoms (Butler et al., 2004). Fatty acids, α-tocopherol, phytosterol, stigmasterol, and campesterol are other constituents of the seed (Pasricha et al., 2014). Faba bean lectin protein has shown binding affinity to HIV-1carbohydrates (François and Balzarini, 2012). Traditionally, cooked faba beans have been applied against cough and inflammation (Prabhu and Rajeswari, 2018). Owing to rich content of phenolic compounds, the seeds have antioxidant property (Pasricha et al., 2014). A study revealed that Leucoefdin found in *Vicia faba* has the potential to inhibit M^Pro^ protease, which is responsible for the formation of functional viral polyprotein (Singh and Mishra, 2020). It is suggested that *Vicia faba* may help to fight better against coronavirus 2 infection (Figure 1) (Khalil et al., 2020). The two other ingredients of purging cassia laooq including *A. gummifera* gum and *P. dulcis* oil are discussed in seeds Laooq and almond Morabba sections, respectively.

#### Seeds Laooq

Seeds Laooq is made of *Astracantha gummifera* (Labill.) Podlech (Leguminosae) gum (moderate and wet temperament), *Cucumis sativus* L. (Cucurbitaceae) seed (cold and wet temperament), *Cucumis melo* L. (Cucurbitaceae) seed (hot and wet temperament), *Cucurbita pepo* L. (Cucurbitaceae) seed (cold and wet temperament), *Prunus dulcis* (Mill.) D.A.Webb (Rosaceae) fruit (hot and wet temperament), sugar (hot and dry temperament), and honey (hot and dry temperament). It has traditionally been recommended for dry cough (Ghaeni Heravi, 1766). Gum tragacanth (*Astracantha gummifera* (Labill.) Podlech) as an adhesive agent and a thickener has a wide range of usage in food and pharmaceutical industries. It is a complex mixture of various polysaccharides acting as laxative and antitussive (Noreen et al., 2019; Fattahi et al., 2013). A water-swellable polysaccharide (bassorin) and a pectic polymer (tragacanthic acid) are available in gum tragacanth (Nayeb morad, et al., 2018; Stephen and Phillips, 2006). Inhalation of *A. gummifera* 2.5% w/v and 5%w/v decreased significantly the number of coughs induced by chemicals in animals (Saadat et al., 2018). *Cucumis melo* L. seeds, possessing dietary fibers, minerals, and antioxidants such as β-carotene, are a valuable source of nutrients with different medicinal indications such as digestive, antitussive, and demulcent (Ibrahim et al., 2019). Melon seed oil containing linoleic acid, lecithin, and cephalin acts as an antimicrobial, antioxidant, and anti-inflammatory agent (Simona et al., 2009). Seeds of *C. sativus* have been traditionally used against fevers and burning sensations (Seliya and Patel, 2009). Ethanolic extract of *C. sativus* seed contains flavonoids, phenols, carbohydrates, terpenoids, and tannins (Begum et al., 2019). *Cucurbita pepo* seed contains amino acids, phenolic compounds, phytosterols, tocopherols, cucurbitacins, minerals, and unsaturated fatty acids such as oleic and linoleic acids (Dotto and Chacha, 2020). Cucurbitacin E isolated from pumpkin seed has shown anti-inflammatory characteristics (Jang et al., 2008). *Prunus* gums are hydrophilic carbohydrates with high molecular weights. They are composed of monosaccharide units linked by glucosidic bonds (Bouaziz et al., 2016). Owing to low-toxicity, stability, and availability, the gums are applied in pharmaceutical industries as an emulsifying agent, disintegrant, suspending agent, and binder (Figure 1) (Rahim et al., 2018).

### Morabba

Morabba has been a popular FF in TPM and the word “Morabba” means treated or trained in Persian. The general meaning of “Morabba” is that ingredients should be treated in the process of jam preparation (Dehkhoda, 1999; Ghaeni Heravi, 1766). In other words, Morabba is a traditional FF similar to jam in which the chopped or sliced natural ingredients are treated in a base of honey, grape juice (*doushab*), or syrup (Ghaeni Heravi, 1766).

Natural honey is a common base of cough jams in TPM (Ghaeni Heravi, 1766). It is a natural sweet food material made from nectar of flowers. Honey is composed mainly of glucose and fructose, and containing amino acids, proteins, enzymes, minerals, vitamins, and other minor compounds (Burlando and Cornara 2013). Its phenolic constituents specifically chrysin modulate the oxidative stress and inflammatory conditions. Current biomedical findings have proved the immunomodulatory and respiratory protective effects of honey. Honey and chrysin have shown beneficial effects through affecting total inflammatory cells, eosinophils, macrophages, lymphocytes, neutrophils, p-Akt, IFN-γ level, serum total IL-4, IgE, and IL-13, α-Smooth muscle protein expression, and ERK1/2 pathways. In addition, chrysin has shown therapeutic effects in the lung injury model through the regulation of TNF-α, NAD-dependent deacetylase (SIRT1)/Nrf2, and IL-1β levels, β-glucuronidasem and myeloperoxidase levels, HO-1, MDA, GSH, VCAM-1, ICAM-1, and NF-κBp65 pathways (Talebi et al., 2020). Recently, chrysin is identified as a COVID-19 main protease inhibitor according to *in silico* studies (Lima et al., 2020). Moreover, chrysin has shown *in vitro* inhibitory effects on herpes-virus intracellular replication (Berretta et al., 2020). Other relevant investigations expressed that honey might have antitussive properties with no side effects (Mulholland and Chang, 2009; Werner and Laccourreye, 2011). In a recent study on upper respiratory tract infections, honey has shown higher therapeutic effects than usual care (Abuelgasim et al., 2021). Besides, a Cochrane systematic review has suggested that honey may have better effects than diphenhydramine on suppression of children’s cough (Oduwole et al., 2018). The efficacy of honey in reducing the COVID-19 symptoms in humans is being studied (Matteo et al., 2020). An *in silico* analysis indicates that M pro may be the anti-covid-19 target of flavon, flavonols, and phenolic esters content of honey (Matteo et al., 2020).

Doushab is another sweet base of TPM cough jams. It is a viscous dark brown liquid obtained from *Vitis vinifera* L. juice (Vitaceae) when the whole grape (*V. vinifera*) fruit is cooked, filtered (to separate its seeds and peel), and then concentrated to get doushab (Gharibzahedi, 2018). This concentrated grape juice contains high amounts of glucose and fructose, essential minerals, and polyphenols (Bozkurt et al., 1999; Haas et al., 2018). Flavonoids such as quercetin, isorhamnetin, and kaempferol derivatives are identified in grape fruits (Georgiev et al., 2014). The protective effects of flavonoids on lungs may be due to their antioxidant and anti-inflammatory properties. Their antioxidant activity involves inhibition of nitric oxide synthase and xanthine oxidase as well as direct free-radical scavenging activity. Besides, the mechanisms of action related to anti-inflammatory properties of flavonoids could be described as inhibitory effects on the 5-lipoxygenase and cyclooxygenase pathways in the metabolism of arachidonic acid (Butler et al., 2004). Quercetin has shown important biological activities including anti-inflammatory, antioxidative, and antihistamine actions as well as protective and preventive effects in controlling asthma complications (Cesarone et al., 2019; Derosa et al., 2021). Quercetin as an antiasthmatic, immunomodulatory, and bronchodilatory agent has induced a relaxation effect in tracheal rings and reduced the inflammatory cytokines and eosinophil peroxidase in the lungs according to one *in vivo* study in a murine model of asthma (Oliveira et al., 2015). Also, quercetin has shown protective effects on COVID-19-induced acute kidney injury (Gu et al., 2021). Furthermore, kaempferol has shown central antitussive activities (Huang, et al., 2020a; Huang et al., 2020b). Gallic acid, as one of the important secondary metabolites present in *Vitis vinifera* L. fruits, has exhibited various biological characteristics such as anti-inflammatory, antimicrobial, and antioxidant properties (Arora et al., 2016). A hot water extract of grape peel has presented antiviral (influenza virus) activity in former studies (Bekhit et al., 2011). Procyanidins in grape extract is identified as a potent antiviral agent (Dai et al., 2012). Also, procyanidins revealed potential therapeutic properties against COVID-19 in molecular docking studies (Maroli et al., 2020). Moreover, resveratrol is a flavonol component of grape that has the ability to bind with the ACE2 target site of COVID-19 according to recent *in silico* studies (Matteo et al., 2020). Resveratrol has antioxidant and immune-stimulatory properties (Ramdani and Bachari, 2020; Santos et al., 2021). Also, by virtue of its anti-inflammatory and anti-thrombotic properties, resveratrol is expected to lower the mortality rate of COVID-19 disease (Giordo et al., 2021).

Based on recent clinical trials on cough formulations, researchers have proposed that there should be one or more characteristics having some physiological effects in the base of cough formulations. For instance, most liquid antitussive preparations are very sweet. Thus, the researchers have suggested that sweet taste may be able to modulate cough sensitivity. According to current evidence-based studies, a close anatomical relation in the brainstem is found between the mechanisms involved in the cough reflex and those processing taste signals. Therefore, primary taste afferents might be responsible for modulation of activity patterns in the brainstem networks controlling airway protective behaviors (Wise et al., 2014). Using honey as a cough remedy throughout history is accordant with this idea. Evidence-based studies have shown that mouth rinsing with the solution of sweet sucrose could increase the cough thresholds. The basic mechanisms related to cough suppression by the sweet taste are not found yet. However, surviving data indicate the potential effects of taste on modulation of cough sensitivity would be a promising issue for further investigations (Wise et al., 2014).

#### Almond Morabba

**FIGURE 2:**
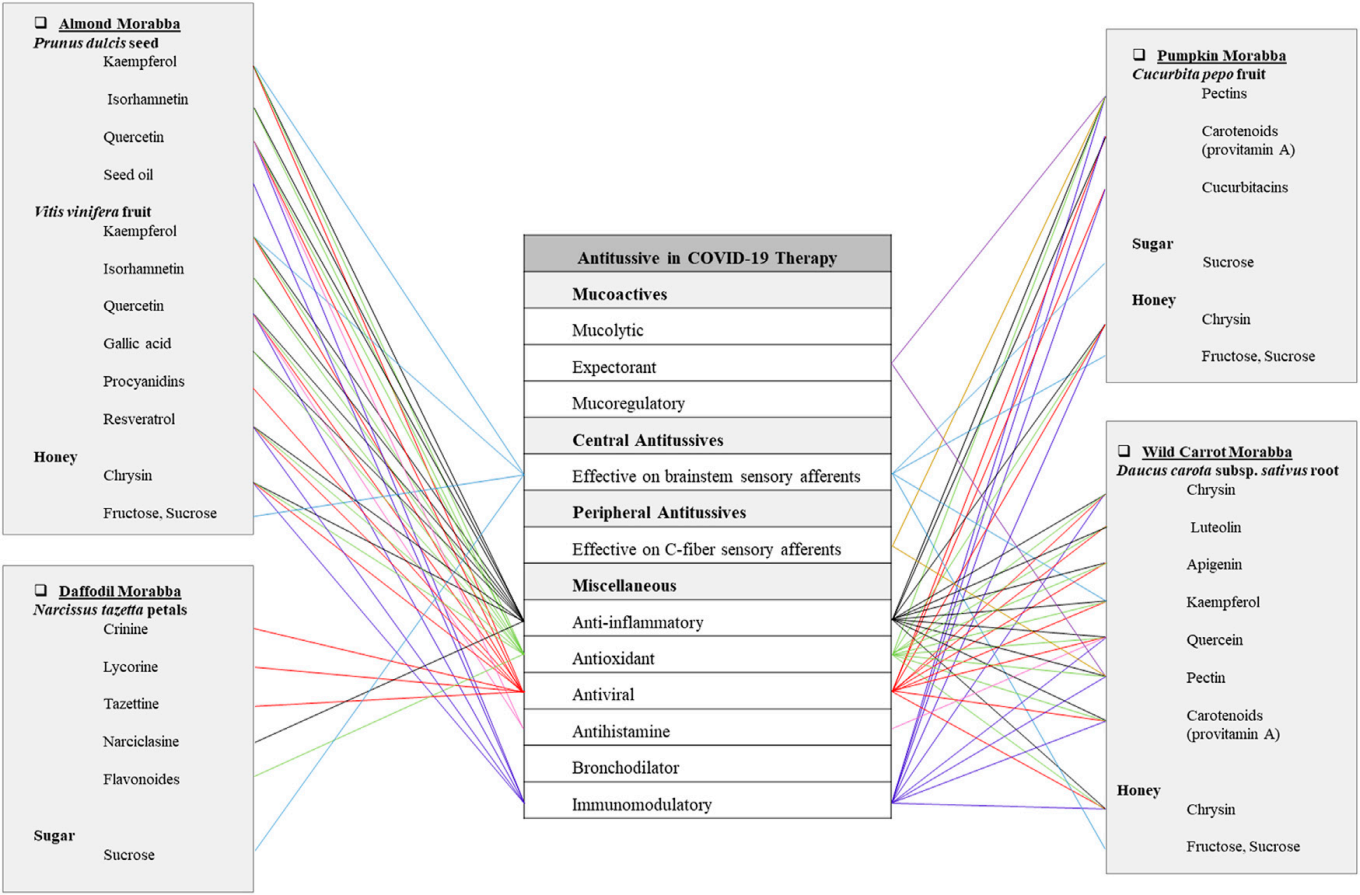
Potentials of antitussive Persian Morabbas in COVID-19 therapy.

Almond Morabba is made of *Prunus dulcis* (Mill.) D.A.Webb (Rosaceae) kernel (hot and wet temperament), Dushab (*Vitis vinifera* L.) (Vitaceae) (hot and wet temperament), and honey (hot and dry temperament). It has been traditionally recommended for dry cough. To prepare this formulation, ripe almond kernel is peeled and boiled with the mixture of water and Dushab (grape juice). Then almond is macerated in that liquid for three days. On the third day, the macerated almond is put in honey and boiled till the mixture gets consolidated. It is kept for 40 days and then becomes ready for taking. It is considered to be suitable for cough and wheezing in TPM (Ghaeni Heravi, 1766). Sweet almond is rich in fatty acids, carbohydrates, proteins, vitamins (vitamin E, B, etc.), minerals, and various bioactive ingredients (polyphenols, phytosterols, etc.) which are consumed as natural anti-inflammatory, antioxidant, antimicrobial, and antiviral agents (Barreca et al., 2020). Different classes of flavonoids including anthocyanidins (cyanidin), flavonols, flavanones, and flavan-3-ols are reported in almond. Among the flavan-3-ols, dihydrokaempferol, (−)-epicatechin, and (+)- catechin are the most abundant compounds but gallocatechin gallate, dihydroquercetin, epicatechin gallate, and epicatechin glycoside are also reported. Additionally, the most abundant flavonoid group in almond are flavonols including kaempferol, isorhamnetin, quercetin, isorhamnetin and their rutinosides, 3-O-glucosides, and galactosides. Also, the main stilbene compound in almond is identified as resveratrol-3-O-glucoside (Barreca et al., 2020). Actually, almond is beneficial to improve the immune system (Ali and Alharbi 2020). The phenolic content of the nuts may decrease or even prevent the processes of oxidative stress-related disorders (Isfahlan et al., 2010; Alkhatib, 2020; Subhashinee et al., 2006). Also, Almond oil can be helpful for the improvement of the immune system and the prevention of many degenerative diseases (Kostadinović Veličkovska et al., 2018). On the other hand, almond kernel has shown prebiotic properties (Gibson and Roberfroid, 1995) while probiotics are supposed to be useful in the management of COVID-19 infection (Figure 2) (Giannoni et al., 2020).

#### Daffodil Morabba

This Morabba is made of *Narcissus tazetta* L. (Amaryllidaceae) petals (hot and dry temperament) and sugar (hot and dry temperament). It has been traditionally recommended for cough and dyspnea. To prepare this TPM formulation, daffodil petals and sugar are mixed and made into jam. According to Persian medicine, it is considered to be suitable for special respiratory disorders. Daffodil contains alkaloids such as crinine, lycorine, and tazettine that presented *in vitro* antiviral and antimalarial activities (Kornienko and Evidente, 2008). Lycorine is a broad-spectrum antiviral substance against coronavirus infection and it is possible to have therapeutic effects in COVID-19 infection (Choudhry et al., 2020; Khalifa et al., 2020). Narciclasine is another alkaloid isolated from the bulbs of different varieties of *Narcissus*. It has significantly reversed the gene expression changes in a moexipril-treated HCC515 cell line which is a suggested model for human lung injury in COVID-19. Also, narciclasine has shown *in vivo* anti-inflammatory effects and lung injury reduction (Kornienko and Evidente, 2008; He and Garmire, 2020). Moreover, some flavan derivatives, β-coumaranone and phenylpropanoid structures with potent *in vitro* antioxidant activity, and a mannose-binding lectin with potent antiviral activity have been isolated from the bulbs of *N. tazetta* (Ooi et al., 2010; Fu et al., 2016). These findings indicate that daffodil may have beneficial effects in COVID-19 (Figure 2).

#### Pumpkin Morabba

Pumpkin Morabba is made of *Cucurbita pepo* L. (Cucurbitaceae) fruit (cold and wet temperament), honey (hot and dry temperament), and sugar (hot and dry temperament). It has been traditionally recommended as a lung-protective FF. The peeled pumpkin is chopped and boiled in water till it becomes semi-cooked, then honey and sugar are added, and the mixture is boiled more till it gets consolidated. Pumpkin contains minerals as calcium, phosphorous, iron, sodium, and potassium. Also, it contains vitamins such as vitamin A, thiamin, riboflavin, niacin, and ascorbic acid (Fernández-López et al., 2020). Pumpkin fruit contains various water-soluble pectins with antitussive efficacy equal to and even more than codeine. Furthermore, pectins did not show any adverse effects in one *in vivo* study. So, it declares that they are safer than conventional opioid cough suppressants (Nosáľová et al., 2011). Pectic polysaccharides are rich in galactopyranosyluronic acid (GalpA), and galactans are polysaccharides with high proportion of galactose (Ferreira et al., 2015). After oral administration of pectins, they can cover the mucus terminals in the epipharyngeal nerve and decrease the sensitivity of cough receptors to irritations which leads to cough suppression. Also, it is considered that these kinds of polysaccharides can increase the saliva production contributed to antitussive properties by the activation of the swallow reflex that is competing with cough reflex in the central level (Nosalova et al., 2013). Pectins have immunomodulating, anti-inflammatory, and antitussive properties. In a recent study, in which cough reflex was induced by citric acid in guinea pigs, pectins from *C. pepo* inhibited the frequency and intensity of coughing attacks. In another study, pectins from the *Althaea officinalis* L., stimulated the activity of airway mucus and the peristalsis of respiratory bronchioles collaborated with the enhanced bronchial glands secretion (Zaitseva et al., 2020). In addition to antitussive properties, pectin fractions of pumpkin have shown antioxidant effects (Torkova et al., 2018). Furthermore, pumpkin has some triterpenes such as cucurbitacins, and some tetraterpenes such as carotenoids. Carotenoids have pro-vitamin A and immunomodulatory activity. Also, pumpkin has antioxidant substances such as β-carotene, lutein, and zeaxanthin that can improve the immune system function (Montesano et al., 2018; Swamy, 2020). According to a recent study, a heat treatment on raw pumpkin could significantly increase the bioaccessibility of its β-carotene compounds (Thakur et al., 2020). On the other hand, it is notable that in one *in silico* study, cucurbitacins (including cucurbitacin E and B, and isocucurbitacin B) represented strong binding affinity to the main protease of COVID-19 that leads to blockage of the COVID-19 viral replication. On the other hand, in recent studies, cucurbitacin B and E showed immune-enhancing activities against HSV-1 and BVDV/HIV, respectively, and they did not show any side effects (Figure 2) (Alagu Lakshmi et al., 2020).

#### Wild Carrot Morabba

Wild Carrot Morabba is made of *Daucus carota* subsp. *sativus* (Hoffm.) Arcang. (Apiaceae) root (hot and wet temperament) and honey (hot and dry temperament). It has been traditionally recommended for antitussive purposes. To prepare this jam, wild carrot is boiled in water containing honey. Then it is placed in another pot and boiled with honey gently until it loses its water. Wild carrot contains flavonoids from flavones group such as apigenin, chrysin, and luteolin, flavonols group such as kaempferol and quercetin, and various glycosides. In addition, the carrot plant has furanocoumarins of 8-methoxypsoralen and 5-methoxypsoralen. Other constituents are choline, daucine (alkaloid), fatty acids (butyric, palmitic), pectins, and coumarins (Barnes et al., 2013; Jafari et al., 2017). Cholinergic actions have been reported from *in vitro* studies, indicating the spasmodic properties of wild carrot in both smooth and skeletal muscle. This cholinergic activity has been attributed to its choline content (Barnes et al., 2013). On the other hand, chrysin is reported to have inhibitory effects on COVID-19 main protease and herpes-virus intracellular replication (Lima et al., 2020; Berretta et al., 2020). While luteolin has the potential to bind to Spike-2 protein, PLpro, M pro/3CLro, and ACE2 (Fuzimoto and Isidoro, 2020; Shawan et al., 2021), apigenin can bind to active residues of ACE2 which intercede host viral interface (Khanna et al., 2021). According to previous studies, *D. carota* herb is a rich source of provitamin A. It is notable that the preparation method of carrot affects its carotenoid content. Water cooking of carrot without any pressure (the same as Persian wild carrot jam recipe) is reported to be the best method for reducing its carotenoids loss (Sant’Ana et al., 1998). Indeed, it is reported that thermal treatment of carrot can have a positive effect on the micellarization of its carotenes as well as disruption of protein-carotenoid complexes in its food matrix and softening its cell wall. This process would significantly improve the bioaccessibility of carotenoids in carrots (Thakur et al., 2020). Furthermore, Bioinformatics findings suggest pharmacological mechanisms for vitamin A against COVID-19 through immunomodulation, anti-inflammatory reaction, and antioxidant properties. Seven core targets of vitamin A against COVID-19, including CAT, EGFR, ICAM1, IL10, MAPK1, MAPK14, and PRKCB, have been detected (Figure 2) (Li R. et al., 2020).

### Saviqs

Saviq is the flour made of roast grains or fruits. According to TPM, Saviq is prepared by a brief roasting process “to the extent that the odor of roasted flour is smelled.” This traditional description may indicate the shortness of heating process to induce a number of modifications such as destruction of microstructures responsible for releasing of the bound phytonutrients (Qi et al., 2018; Thakur et al., 2020). But, the potential nutritional aspects of the flour would not have considerable changes after a short heat treatment (Schnorr et al., 2016; Qi et al., 2018). It is evident that roasted fruits have more antioxidant effects than the raw ones according to recent studies (Navajas-Porras et al., 2020). Saviq is a popular snack in Iran. It has beneficial effects related to its ingredient materials with more astringent characteristics than its raw materials (Shafiee et al., 2019).

#### Pumpkin Saviq

**FIGURE 3:**
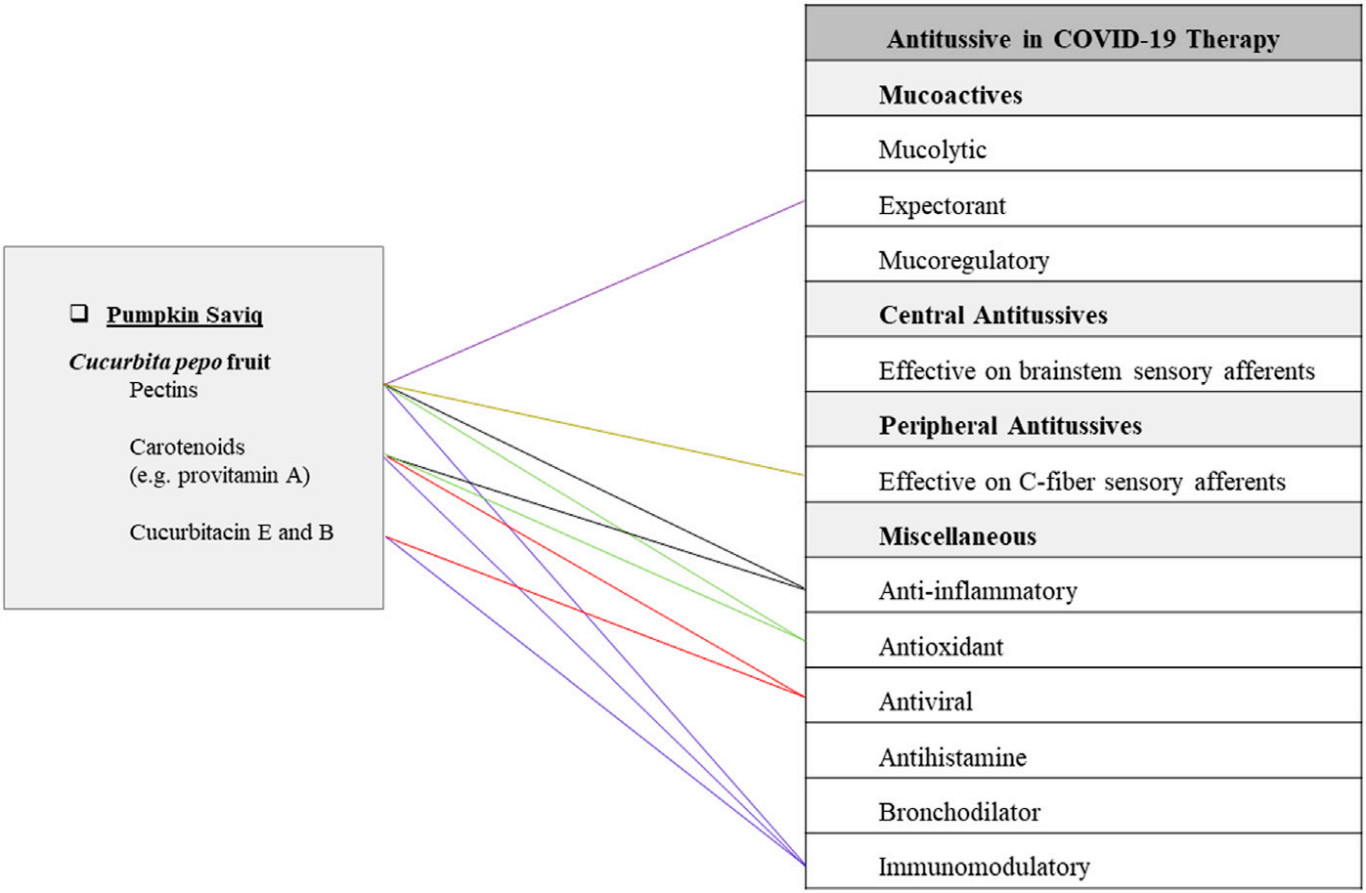
Potentials of pumpkin Saviq as an antitussive Persian functional food in COVID-19 therapy.

It is made of *Cucurbita pepo* L. (Cucurbitaceae) roast fruit (cold and wet temperament). It has been traditionally recommended as a very effective FF for coughs associated with hot distemperament. For preparing pumpkin Saviq, pumpkin is peeled, chopped, dried, and after roasting, grinded to become a fine powder. It is considered to be helpful as an antitussive agent (Ghaeni Heravi, 1766). The current investigations related to pumpkin potential for the treatment of COVID-19 are described in the part pumpkin jam (Figure 3).

### Soups (Shorbas)

Soup is a popular nutritious and flavorful watery food in TPM. It is used in winter and cold weathers especially for the prevention or treatment of common cold and influenza. For Iranians, Shorba is a kind of folk soup which is sometimes salty and spicy (Ghaeni Heravi, 1766). Consumption of a bowl of hot soup and breathing its warm vapors is considered to have mucolytic effects (Kirkpatrick, 1996).

#### Rooster Soup

This soup is made of *Gallus gallus domesticus* (Phasianidae) meat (hot and dry temperament), *Polypodium vulgare* L. (Polypodiaceae) rhizome (hot and dry temperament), *Anethum graveolens* L. (Apiaceae) aerial part (hot and dry temperament), and *Apium graveolens* L. (Apiaceae) aerial part (hot and dry temperament). It has been traditionally recommended for the treatment of phlegmatic cough, pulmonary infection, and dyspnea. According to TPM manuscripts, rooster soup contains a potent active ingredient to cure the respiratory infections, phlegmatic coughs, and dyspnea (Gharashi, 1288). The recipe of this traditional soup is that the rooster is killed and its feathers and visceral content are removed. Then it is stuffed with polypody, dill, and celery and further it is boiled till the meat of rooster crushes and it is filtered to prepare a soup (Ghaeni Heravi, 1766). It is known that soft connective tissue and the comb of rooster are good sources of hyaluronic acid (HA). Indeed, the rooster comb has the highest concentrations of HA among animal tissues (Boeriu et al., 2013). Thus, an important active ingredient of this traditional soup seems to be HA, which is an extracellular matrix polysaccharide with the ability of trapping water to produce a hydrogel substance (Swann, 1968; Garvin et al., 2020). In fact, HA is a carbohydrate compound with repeating disaccharide units of glucosamine and N-acetylglucosamine (Ferreira et al., 2015). HA, as a lubricant at the epithelium surface of airway, can increase the intercellular function of adhesion molecules in airway mucus by modulating its surface properties and improving the surface activities of the respiratory tract surfactants. Moreover, HA strengthens the bronchial epithelial barrier, stimulates the cellular host defense mechanisms, and increases the ciliary beating in mucosal host defense. Besides, HA is associated with protective mechanisms against cell death including the interactions of HLA-Toll-like receptor, basal activation of NF-κB, and specific interactions with several cell surface receptors such as CD44. Generally, HA is considered a protective factor in various cell aggressions against the airway epithelium and the epithelial integrity homeostasis. Also, it is a lubricant agent supporting the good ciliary and cough clearance in the airway mucus (Zahm et al., 2011). HA has relevant interactions with immune cells. In a normal lung, alveolar macrophages are surrounded by a HA layer. In acute lung infection, HA levels are immediately increased suggesting a potential role for HA in the promotion of leukocyte accessibility to the lung injury site. Additionally, HA can produce a provisional matrix to promote tissue repair (Johnson et al., 2018). Notably, it is evident that in lungs of COVID-19 patients, the production of HA is increased and its degrading enzyme is greatly decreased. In addition, the levels of ACE2 and bradykinin in their lung cells are increased. These changes trigger leakage of fluid into the patient's lungs and the produced HA combines to that fluid. It results in a hydrogel formation that prevents gas exchange in the lungs and it leads to a drastic condition in severe COVID-19 patients (Garvin et al., 2020). According to recent investigations, alveolar HA level is usually elevated in lung injuries, while aerosolized HA has shown healing effects in lung diseases associated with elastic fiber injury. It is reported that the larger HA molecules possess anti-inflammatory properties and smaller molecules have pro-inflammatory activities (Noble et al., 2011; Esposito et al., 2017). Thus, there is a size-effect relationship regarding hyaluronan molecules. Those with a molecular weight of 1,050–1,338 kDa have shown stimulatory activity on various immune cells in recent studies. Also, those with the molecular weight of 45.2–145 kDa have shown stronger immunostimulatory activity after the process of hydrolysis (Ferreira et al., 2015). A recent study suggested that the administration of exogenous HA by aerosol could have therapeutic effects on diseases in which their exacerbation alters the surface properties of the mucus and the mucucilliary clearance functions. Another study revealed that HA 40 kDa protected the airway epithelium against the injury during bacterial infection (Zahm et al., 2011). Moreover, HA has antioxidant activity and it regulates inflammatory cell recruitment, inflammatory cytokines release, and stem cell migration in inflammation and tissue injuries (Noble et al., 2011; Hafsa et al., 2017). These findings provide a new insight on the probability of using HA molecule to manage COVID-19 respiratory symptoms. On the other hand, rooster meat, as another ingredient of this soup, is a rich source of immunomodulatory peptides, vitamins, and minerals that can increase the immunity of body against coronavirus by enhancing of macrophages and monocytes functions (Alkhatib, 2020).

Polypody (*Polypodium vulgare* L.) is another ingredient of the traditional rooster soup. Polipody contains flavonoids such as kaempferol and quercetin derivatives, as well as hydroxycinnamic acids including caffeic acid derivatives and chlorogenic acid. Also, it has phytoecdysteroids such as 20-hydroxyecdysone and polypodin B, steroidal saponins such as osladin and polypodosaponins. Furthermore, numerous triterpenoids comprising cuphan, cycloartane, dammaran, and phernan, and some other phytochemicals such as cycloartanyl acetate, cycloaudenyl acetate, linoleic acid esters, and phytosterols are reported in polypody extracts. Pharmacological and clinical studies on *P. vulgare* are rare (Barnes et al., 2013), but recent studies on *P. leucotomos* Poir. phenolic compounds (e.g., chlorogenic, coumaric, vanillic, caffeic, and ferulic acids) have demonstrated antioxidant properties through *in vitro*, *in vivo*, and human studies (Berman et al., 2016). In a recent clinical trial, *P. leucotomos* extract prevented the infection processes in athletes by enhancing their immune system. Besides, *in vitro* studies on polypody extract have demonstrated its pleiotropic effect on different cytokines of the immune system. In fact, *P. leucotomos* has displayed both humoral and cellular immunomodulatory activities through *in vitro* studies (Solivellas and Martin, 2012; Sánchez-Rodríguez et al., 2018). On the other hand, polypody expressed healing properties in the treatment of tyrosine kinase-induced phototoxicity in a case report study (Korman et al., 2019). It is noteworthy that a recent bioinformatics analysis has suggested that any herb with the anti-tyrosine kinase activity could be a good drug candidate for treating COVID-19 infection (Sriwijitalai and Wiwanitkit, 2020).

Dill (*Anethum graveolens* L.) is another ingredient of TPM rooster soup. It has two major flavonoids including isorhamnetin 3-O-β-d-glucuronide and quercetin 3-O-β-d-glucuronide, as well as other minor components including 3-glucosides, 3-galactosides, and 3-rhamnoglucosides of quercetin and isorhamnetin. Volatile components of dill include carvone, limonene, α-phellandrene, dill ether (anethofuran), coumarins, myristicin, flavonoids, steroids, and phenolic acids. 8-hydroxygeraniol, β-d-glucopyranosides, and p-menth-2-ene-1,6-diol have also been isolated from the dill herb. A furanocoumarin, several coumarin derivatives, phenolic acids such as caffeic, ferulic, and chlorogenic acids are detected in dill seeds. Major constituents of hydro distilled essential oil from aerial parts of Persian dill are limonene, α-phellandrene, dill ether, and sabinene. Limonene and sabinene have been identified as its main antioxidant compounds (Akbar, 2020). Dill also contains carotenoids, ascorbic acid, and minerals (Naidu et al., 2016). Oral indication of dill (aqueous extract), in one *in vivo* study, showed potential antioxidant properties (Oshaghi et al., 2016). Generally, vegetables are known as FFs that can prevent and control viral infections by inducing antioxidant and anti-inflammation activities to modulate the immune system (Alkhatib, 2020). Also, coumarins have shown strong antioxidant activities (Shekhar et al., 2017). It is believed that the water-soluble antioxidants can protect lipid-soluble antioxidants via a polar paradox (Nayak et al., 2015). Interactions between the matrices of soup vegetables and the lipid fractions during cooking are also notable.

**FIGURE 4:**
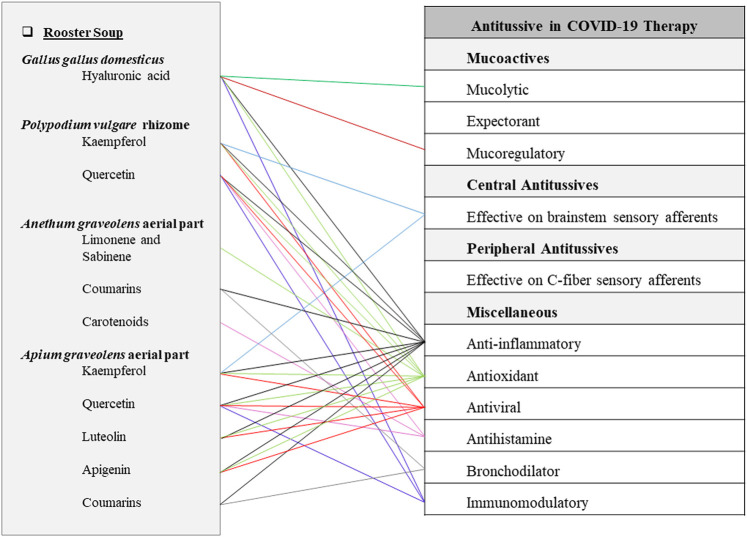
Potentials of rooster soup as an antitussive Persian functional food in COVID-19 therapy.

Celery (*Apium graveolens* L.), another ingredient of rooster soup, possesses flavonoids including apigenin, apiin, quercetin, isoquercitrin, kaempferol, and luteolin, coumarins including apigravin, apiumoside, apiumetin, bergapten, celereoside, celerin, isoimperatorin, isopimpinellin, osthenol, umbelliferone, rutaretin, seselin, and 8-hydroxy-5-methoxypsoralen, and fatty acids, caffeic, p-coumaric, and ferulic acids (Barnes et al., 2013; Kooti and Daraei, 2017; Chonpathompikunlert et al., 2018; Li M. Y. et al., 2020). It has powerful antioxidant properties due to its phytochemical compounds such as caffeic, p-coumaric, and ferulic acids, apigenin, kaempferol, luteolin, quercetin, saponin, and tannin. In particular, celery has more apigenin content compared with other plants (Kooti and Daraei, 2017; Chonpathompikunlert et al., 2018; Li M. Y. et al., 2020). Additionally, celery plant extracts have shown anti-inflammatory activities *in vitro* and *in vivo* (Barnes et al., 2013; Akbar, 2020). Besides, one teaspoon of celery seeds mixed with foods, taken three times a day, has shown beneficial effects in chest pain, asthma, and bronchitis in recent investigations. Its coumarins are supposed to have the muscle relaxant activities, as well as its antispasmodic property that is pertained to the essential oil in seeds (Peter, 2012). Apigenin, luteolin, kaempferol, and quercetin have antioxidant and anti-inflammatory activities (Tian et al., 2021). Furthermore, flavonoid compounds such as apigenin, kaempferol, and quercetin showed activity against COVID-19 through suppression of M pro enzymes. Also, the target for apigenin is considered to be spike protein, 6LU7, and 6Y2E proteases (Bhuiyan et al., 2020; Matteo et al., 2020). In addition, luteolin can bind to Spike-2 protein of SARS-CoV without any cytotoxic effects (Figure 4) (Fuzimoto and Isidoro, 2020).

### Syrups

Syrup is a well-known liquid dosage form in both traditional and modern medicine. Sweet violet (*Viola odorata* L.) syrup has been one of the most popular TPM drinks recommended for antitussive properties (Ghaeni Heravi, 1766). According to clinical and *in vivo* studies, the syrupy vehicle of antitussive syrups possesses a local demulcent effect on the mucosa of respiratory tract and relieves the irritation of cough in the mucosa. Also, drinking syrup might have little expectorant property when the bulk of syrup enters into the stomach. These experiments provide a logical reason for traditional application of antitussives in a vehicle of syrup, and also justify the persist use of this form of medication (Boyd, 1946). This syrup has been recommended for the prevention or management of epidemic situations such as COVID-19 in the ancient times (Ansari et al., 2020).

#### Sweet Violet Syrup

**FIGURE 5:**
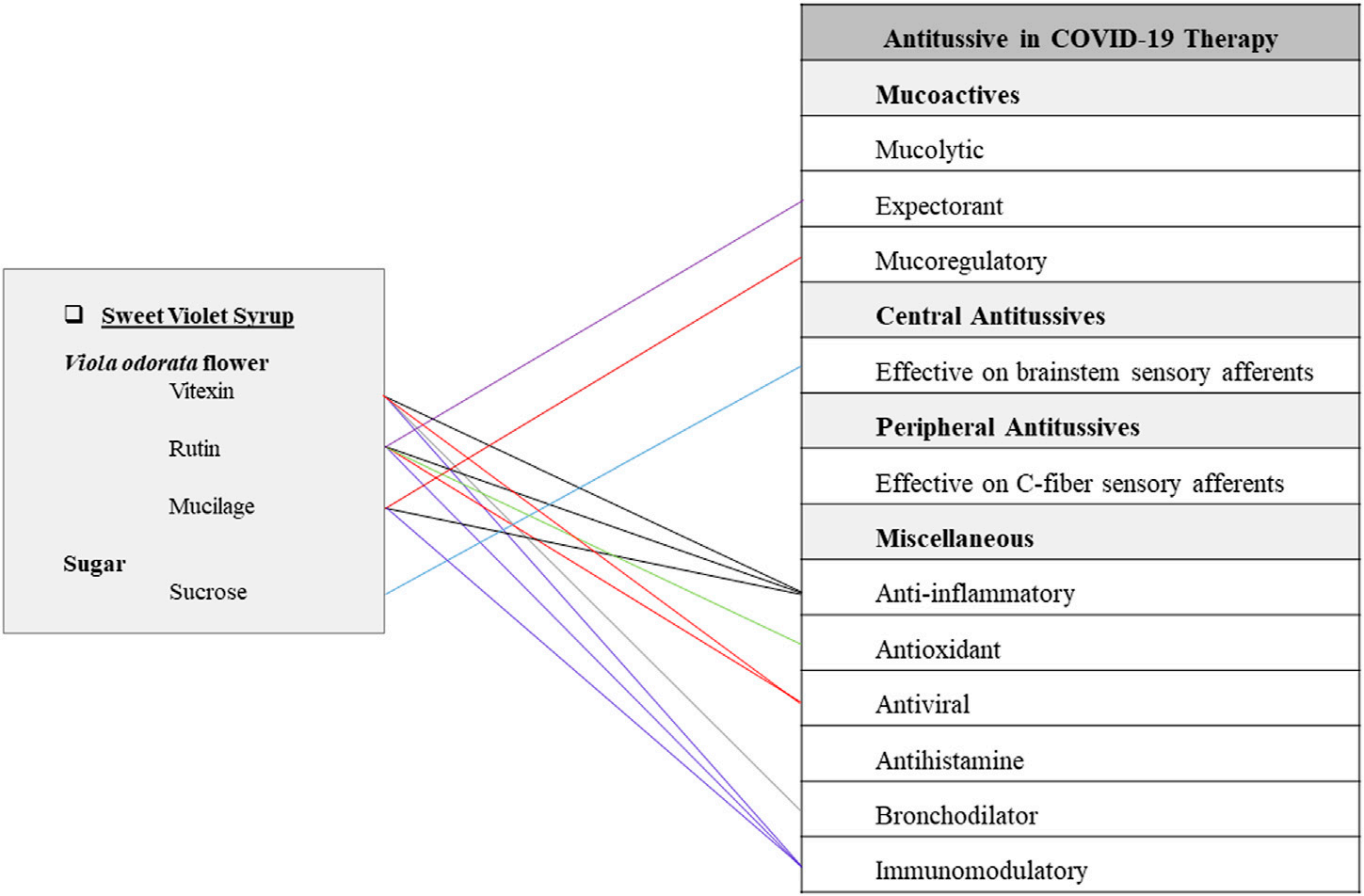
Potentials of sweet violet syrup as an antitussive Persian functional food in COVID-19 therapy.

This syrup is made of *Viola odorata* L. (Violaceae) flower (cold and wet temperament) and sugar (hot and dry temperament). It has been traditionally recommended for dry cough, respiratory infections, and fever. To prepare this Persian FF, sweet violet flower is boiled in water till two-thirds of the water evaporates. The residue of petals should be strained and filtered. Then sugar is added to the liquid and the syrup is boiled to be more concentrated (Ghaeni Heravi, 1766). Sweet violet flower contains mucilage, anthocyanins, flavonoids, and methyl salicylate (Tobyn et al., 2011). Aqueous-methanol leaves extract possesses alkaloids, coumarins, tannins, phenolics, flavonoids, and saponins. Aqueous extract contains vitexin while the ethanol extract contains vitexin, isovitexin, rutin, and kaempferol-6-glucoside (Akbar, 2020). *V. odorata* is a rich source of antioxidants (Mousavi et al., 2016). Vitexin has shown decreasing effects in lung edema and alveolar protein content. An *in vivo* study revealed that vitexin could suppress neutrophil recruitment and proinflammatory cytokine levels, but increase Nrf2 and HO-1 activity. These findings expressed that vitexin could suppress LPS-induced acute lung injury by controlling the Nrf2 pathway (Lu et al., 2018). On the other hand, vitexin has shown spasmolytic effects on rat-isolated duodenums by increasing cGMP and activating K+-channels (Ragone et al., 2007). According to *in-silico* virtual studies, vitexin has potential inhibitory effects on spike protein and 3CLpro or M pro of COVID-19 virus (Naik et al., 2021). Furthermore, rutin has shown *in vivo* antiasthmatic activity by decreasing eosinophils and neutrophils in lung (Ganeshpurkar and Saluja, 2017). Evidence from current studies has shown that sweet violet successfully has treated the children’s cough (Qasemzadeh et al., 2015). Also, sweet violet has shown anti-inflammatory, anti-asthmatic, analgesic, anti-microbial, antipyretic, and lung tissue protecting characteristics in current studies (Yazdi et al., 2020; Arsley et al., 2018; Muhammad et al., 2012). Demulcent effect of mucilage compounds in this herb could be beneficial for healing oral and pharyngeal irritations or other related complications such as throat pain, throat scratching, dry cough, and difficulty in swallowing (Figure 5) (Kheterpal et al., 1989; Brinckmann et al., 2003).

## Discussion and Conclusion

TPM that is the heritage of prominent traditional Persian physicians has been long and widely used in the prevention and treatment of various difficult miscellaneous diseases by virtue of its abundant sources, diverse structures, and novel activities. Also, local traditional herbs are the source of clues or inspirations for the scientists in the field of drug discovery. TPM has classified practical medicine into hygiene and therapeutic medicine branches. It has had focused specially on FFs benefits to human health. The significance of FFs in TPM view is so much that the great Iranian scientist, Rhazes, said: “When you can use foods for treatment of diseases, avoid medicaments.” On the other hand, the memory background and nutrient nature of FFs might evoke better compliance in patients and better therapeutic outcomes (Amiri Ardekani et al., 2020). Therefore, TPM FFs would be beneficial natural supplements and potential candidates for COVID-19 therapy. Cough, as a pathological reflex, is a common symptom of SARS-CoV-2 infection. In the present study, a review has been done on possible protective and therapeutic pharmacological mechanisms of antitussive TPM FFs against COVID-19 with the notice that elimination of cough is an imperative issue to decrease transmission of the SARS-CoV-2 pathogen. TPM FFs are made of safe edible natural compounds which are included in the daily diet of people all around the world. In this regard, FFs would be favorable medical agents to combat the current global pandemic. Moreover, FFs could be consumed as protective agents by healthy people and ones who are prone to get COVID-19. An advantage of providing TPM FF formulations is that their preparation method and other required information about their indication and dose of administration are cited in TPM manuscripts based on traditional experiments. In this study, 12 TPM FF formulations were introduced which include 5 Laooqs (garlic, linseed, pomegranate, purging cassia, seeds Laooqs), 4 Morabbas (almond, daffodil, pumpkin, and wild carrot Morabbas), 1 Saviq (pumpkin Saviq), 1 soup (rooster soup), and 1 syrup (sweet violet syrup) formulation. They are combinations of 2–7 ingredients. The various FF types affect the bioavailability of their phytonutrients through changing the microstructural elements such as cell walls, starch granules, and proteins as well as the physical state of their raw materials (Parada and Aguilera, 2007; Marze, 2017). For instance, syrups and Morabbas usually undergo a cooking process in which the bioaccessiblility of the nutrients is increased because of the structural changes responsible for releasing the bound compounds in the food matrices (Thakur et al., 2020). Some phytochemicals such as soluble antioxidants may be destroyed in heating treatment but not those that are bound in the matrices (Parada and Aguilera, 2007). In this context, it is suggested that the complex mixtures of phytochemicals in FFs are causing strong health benefits because of their synergistic and/or additive effects (Nayak et al., 2015).

This article provides a phytochemical approach for using traditional Persian antitussive FFs to combat COVID-19. The most prevalent secondary metabolites among 22 natural ingredients of mentioned TPM FFs are quercetin and kaempferol which are present in 5 plant sources, while apigenin, isorhamnetin, luteolin, and rutin are prevalent in 2 plant sources. It is worth nothing that both kaempferol and quercetin have shown potential direct inhibitory effects on 3CLpro and PLpro, two enzymes that are required for the replication of SARS-CoV-2 virus according to recent studies (Zhang et al., 2020). Some of the chemical compounds contained in the mentioned FFs are found to be multifunction (Figures 1–5). For instance, rutin has anti-inflammatory, antioxidant, antiviral, immunomodulatory, and expectorant characteristics. Also, quercetin has anti-inflammatory, antioxidant, antihistamine, antiviral, and immunomodulatory properties (Figure 1). In addition, pectins are anti-inflammatory, antioxidant, immunomodulatory, expectorant, and peripheral antitussive substances (Figure 2). Furthermore, HA has anti-inflammatory, antioxidant, immunomodulatory, mucolytic, and mucoregulatory properties (Figure 4). Therefore, the main ingredients of the mentioned TPM FFs are predicted to have potential pharmacological benefits against COVID-19. Some of the mentioned components are prevalent in several plants. For example, flavonoids such as apigenin, kaempferol, luteolin, and quercetin are found in many plant species. Some other molecules are more infrequently found in the nature: for instance, vitexin in sweet violet flower which has decreasing effects in lung edema and alveolar protein content (Lu et al., 2018), or lycorine in daffodil which is a broad-spectrum antiviral substance against human coronaviruses infection (Choudhry et al., 2020; Khalifa et al., 2020). Generally, natural constituents of the mentioned formulations have main pharmacological mechanisms including mucoactive functions by expectorant, mucolytic, and mucoregulatory activities as well as central and peripheral antitussives effects, anti-inflammatory, antioxidant, antiviral, antihistamine, bronchodilator, and immunomodulatory effects.

Laooq, made of powdered natural plants in a viscous syrup or honey, is a popular TPM formulation with local demulcent and antitussive effects. High viscosity of Laooq during licking leads to its longer transit time through esophagus (Iwu et al., 2009; Zarshenas et al., 2013). Sugars used in Laooqs, Morabbas, and syrups have sweetening and texturizing effects (Bayarri et al., 2004). TPM has described special mechanisms of action for each compound formulation based on humoral doctrine. For instance, sugar is considered to have a hot and dry nature with muco-protective and emollient properties on the respiratory tract and lungs according to TPM. Also, it could help clear away pulmonary toxins and infections (Gharashi, 1288). This traditional point of view is in consent with modern investigations. Syrup-based formulations and oral solution of sucrose have shown demulcent effects on the irritated mucosa of pharynx and cough suppressive effects in evidence-based studies (Boyd, 1946; Wise et al., 2014). On the other hand, chrysin present in honey has antioxidant, anti-inflammatory, antiviral, immunomodulatory, and respiratory protective effects through regulating total inflammatory cells, eosinophils, macrophages, lymphocytes, neutrophils, p-Akt, IgE, serum total IL-4, IL-13, IFN-γ level, α-Smooth muscle protein expression, TNF-α, NAD-dependent deacetylase (SIRT1)/Nrf2, IL-1β levels, HO-1, MDA, GSH, VCAM-1, ICAM-1, and ERK1/2, β-glucuronidasem and myeloperoxidase levels, and NF-κBp65 pathways (Talebi et al., 2020). Recently, chrysin is identified as a COVID-19 main protease inhibitor according to *in silico* studies (Lima et al., 2020). Moreover, flavon, flavonols, and phenolic esters content of honey have shown *in silico* inhibitory effects on M pro of COVID-19 (Matteo et al., 2020). Honey is considered a hot and dry agent with potent antitussive and emollient characteristics responsible for clearing away toxins in the respiratory tract according to TPM (Gharashi, 1288). Another traditional mechanism of action in Laooqs is seen in *P. dulcis* which is a hot and wet substance suppressing cough and eliminating dampness in the lungs. Additionally, fatty acids in oily seeds such as *L. usitatissimum*, *C. sativus*, *C. melo*, *C. pepo*, and *P. dulcis* are demulcent agents that form a soothing film over mucus membranes. Through mucoprotection, the existing inflammation in the respiratory system decreases. Cold-temperament materia medica can balance the heat resulting from fever and inflammation. Seeds of *C. sativus* and *C. melo*, and flower of *V. odorata*, have an important role in controlling high body temperature. Based on TPM, dry cough and fever are related ailments to bile with hot and dry quality. Therefore, fruits and herbs that remove extra bile from the body can improve the condition. Fruits of *P. granatum*, and *C. fistula* are two ingredients effective on bilious disorders (Badr and Sardari, 2019). According to our results, garlic Laooq, traditionally recommended as a phlegm remover from lungs in TPM, has remarkable anti-inflammatory, antiviral, and immunomodulatory properties. Linseed Laooq, as a TPM FF for dry cough, has notable mucoregulatory and immunomodulatory effects. Pomegranate Laooq, traditionally recommended for cough associated with hot dis-temperament (infection), has notable anti-inflammatory and immunomodulatory properties. Purging cassia Laooq, as traditional FF for pulmonary infection and cough, has remarkable expectorant and anti-inflammatory effects. Furthermore, seeds Laooq, traditionally known as a dry cough remedy, has significant anti-inflammatory, immunomodulatory, and expectorant properties (Figure 1).

Morabba, another popular food in Iran, is made of the treated chopped fruits, flowers, or other natural ingredients in the base of honey, Doushab (grape juice), or sugar. Doushab is considered to possess hot and wet temperament with lower hotness and higher wetness properties. The nature of Doushab is responsible for its muco-protective and emollient features (Ghaeni Heravi, 1766). It exhibits anti-inflammatory, anti-microbial, and antioxidant properties in current investigations (Arora et al., 2016). Furthermore, procyanidins in Doushab is a potent antiviral agent that revealed possible therapeutic effects against COVID-19 in molecular docking studies (Dai et al., 2012; Maroli et al., 2020). Additionally, resveratrol in Doushab could bind to the ACE2 target site of coronavirus-2 according to recent *in silico* studies (Matteo et al., 2020). Almond Morabba, traditionally indicated as dry cough remedy, has remarkable central antitussive effects as well as anti-inflammatory, immunomodulatory, antiviral, and antihistamine effects. Daffodil Morabba, recommended for cough and dyspnea in TPM, has notable antiviral effects. Pumkin Morraba, as a lung protective FF, has notable anti-inflammatory and immunomodulatory as well as expectorant and peripheral antitussive activities. Wild carrot Morabba, traditionally known as an antitussive FF, has noticeable anti-inflammatory, antiviral, immunomodulatory, as well as expectorant and peripheral antitussive effects (Figure 2).

Saviq, as a popular snack in Iran, possesses various beneficial effects related to its ingredient materials (Shafiee et al., 2019). Pumpkin Saviq is considered a potent antitussive FF in TPM because of its cold and wet nature to provide anti-inflammatory and demulcent properties (Gharashi, 1288). It has been traditionally indicated for hot coughs. According to evidence-based studies, pumpkin Saviq can have anti-inflammatory, antiviral, and immunomodulatory as well as expectorant and peripheral antitussive activities (Figure 3).

Soup, as a well-known nutritious FF in TPM, is prepared by a cooking process that the duration of heating can affect the bioavailability of its nutrients. A recent study demonstrated that high-intensity cooking promotes heat degradation of meet proteins in chicken soup. As the result, water-soluble degradation substances can be released in the soup liquid, triggering an increase in its protein content (Qi et al., 2018). Eating a bowl of hot soup is considered to be very useful for cough and respiratory discomforts due to its mucolytic effects by breathing its warm vapors (Kirkpatrick, 1996). According to the TPM literature, rooster soup is responsible for eliminating dampness and infections in the lung and clearing away the residues. Therefore, it has been recommended for phlegmatic cough, pulmonary infection, and dyspnea. Besides, our study revealed that rooster soup can have remarkable anti-inflammatory, antiviral, antihistamine, and immunomodulatory activities as well as mucoactive and bronchodilator properties according to recent investigations (Figure 4). On the other hand, rooster soup contains HA from which pharmaceutical supplements could be extracted. In the past, HA supplements were extracted directly from rooster comb presented in the TPM rooster soup (Swann, 1968). It is noteworthy that the alveolar HA level is elevated in lung injuries, as well as in COVID-19 infection (Esposito et al., 2017; Garvin et al., 2020). Concurrently, aerosolized HA has shown preventive effects on lung diseases accompanied with elastic fiber injuries (Noble et al., 2011). Taken together, according to the traditional indications and recent studies, we hope that the TPM rooster soup containing HA may bring new insights to treat COVID-19 and its associated pulmonary edema.

Syrup is another popular functional food mentioned in this article. Sweet violet flower that is a cold and wet substance is supposed to induce a moderate coldness along with its wetting properties. This special nature has made *V. odorata* a potent herbal medicine for hot pulmonary disorders (such as infections) and dry respiratory discomforts. Sweet violet syrup, traditionally known as a remedy for dry cough, respiratory infection, and fever, has notable anti-inflammatory, immunomodulatory, and mucoactive properties (Figure 5).

In conclusion, FFs can have beneficial effects against the present viral pandemic. Eating FFs containing flowers, fruits, vegetables, and other edible natural substances, instead of consuming chemical drug dosage forms, might have positive psychological effects on COVID-19 patients. Indeed, organoleptic characteristics of FFs including their appearance, aroma, and taste do not induce the unpleasant feelings of using chemical drug dosage forms. Moreover, FFs could have pharmacological effects along with their high nutritional virtues. TPM FFs can reinforce the body power and enhance the immunity system. Also, FFs are cost-effective, easily accessible, and almost safe formulations for both treatment and prevention of the disease. Acquaintance of people and healthcare providers with Persian medicine FFs can be helpful in this global epidemic and may provide better treatment and prevention options. In addition, since the new concerns have grown about persistent respiratory complications after recovery from COVID-19 and its subsequent community morbidity, substantial management for future public health is required. This review recommends TPM FFs to manage such persistent respiratory discomforts after recovery from COVID-19. On the whole, we can conclude that FFs have co-therapeutic and protective effects against COVID-19 infection. TPM has recommended specific antitussive FFs that are safe even in high doses. The correlation of pharmacological mechanisms of action and the molecules found in the current study revealed that cough symptom, as a common pathological reflex in COVID-19 patients, can be alleviated by TPM FFs. Though the scientific or academic evidences are weak, the knowledge and application of traditional local medicine/FF is really a treasure for the health of local people, especially for the emergency epidemic situation such as COVID-19. And the intention is worth encouraging to find a solution during difficult times to deal with the epidemic. Further studies are suggested to focus on the antitussive mechanism of action and bioaccessibility of nutritional compounds in traditional Persian FFs.

## References

[B1] Abuelgasim H. Albury C. Lee J. (2021). Effectiveness of Honey for Symptomatic Relief in Upper Respiratory Tract Infections: a Systematic Review and Meta-Analysis. Bmj Ebm 26, 57–64. 10.1136/bmjebm-2020-111336 32817011

[B2] Aghili Khorasani M. H. (1771). Makhzan-al-Adviah (Rewritten by Shams Ardakani MR, Rahimi R, Farjadmand F). 1st ed. Tehran: Tehran University of Medical Sciences.

[B3] Akbar Sh. (2020). Handbook of 200 Medicinal Plants: A Comprehensive Review of Their Traditional Medical Uses and Scientific Justifications. Switzerland: Springer Nature Switzerland.

[B4] Alagu Lakshmi S. Shafreen R. M. B. Priya A. Shunmugiah K. P. (2020). Ethnomedicines of Indian Origin for Combating COVID-19 Infection by Hampering the Viral Replication: Using Structure-Based Drug Discovery Approach. J. Biomol. Struct. Dyn. 1, 1–16. 10.1080/07391102.2020.1778537 PMC7332876 32573351

[B5] Alam M. A. Quamri M. A. Sofi G. Ayman U. Ansari S. Ahad M. (2020). Understanding COVID-19 in the Light of Epidemic Disease Described in Unani Medicine. Drug Metab. Pers. Ther. 11, 123. 10.1515/dmdi-2020-0136 32966232

[B6] Ali I. Alharbi O. M. L. (2020). COVID-19: Disease, Management, Treatment, and Social Impact. Sci. Total Environ. 728, 138861. 10.1016/j.scitotenv.2020.138861 32344226 PMC7175909

[B7] Alkhatib A. (2020). Antiviral Functional Foods and Exercise Lifestyle Prevention of Coronavirus. Nutrients 12, 2633. 10.3390/nu12092633 PMC7551447 32872374

[B8] Amiri Ardekani E. Askari H. Mohagheghzadeh A. (2020). Memorial Functional Foods: A New Concept from Bavi Tribe. J. Ethn. Food 7, 9. 10.1186/s42779-020-00046-4

[B9] Ansari A. P. Ahmed N. Z. Ahmed K. K. Khan A. A. (2020). An Insight on Wabāi Amrād (Epidemic Diseases) and COVID-19 like Conditions—Unani Perspective. Ijcrr 12, 109–119. 10.31782/IJCRR.2020.12177

[B10] Antwi A. O. Obiri D. D. Osafo N. (2017). Stigmasterol Modulates Allergic Airway Inflammation in guinea Pig Model of Ovalbumin-Induced Asthma. Mediators Inflamm. 2017, 1–11. 10.1155/2017/2953930 PMC5438858 28555089

[B11] Arora P. Ansari S. H. Najmi A. K. Anjum V. Ahmad S. (2016). Investigation of Anti-asthmatic Potential of Dried Fruits of *Vitis vinifera* L. In Animal Model of Bronchial Asthma. Allergy Asthma. Clin. Immunol. 12, 42. 10.1186/s13223-016-0145-x 27536321 PMC4988050

[B12] Arsley N. C. Kirkpatrick C. L. Crittenden C. M. Rad J. G. Hoskin D. W. Brodbelt J. S. (2018). PepSAVI-MS Reveals Anticancer and Antifungal Cycloviolacins in *Viola Odorata* . Phytochemistry 152, 61–70. 10.1016/j.phytochem.2018.04.014 29734037 PMC6003877

[B13] Avicenna H. (1025). The Canon of Medicine (Qanoon-Fil-Tib). Vol.3 Translated by Sharafkandi. A. Tehran: Soroush Publication.

[B14] Azer S. A. (2020). COVID-19: Pathophysiology, Diagnosis, Complications and Investigational Therapeutics. New Microbes and New Infections 37, 100738. 10.1016/j.nmni.2020.100738 32834902 PMC7403867

[B15] Badr P. Mosleh G. Shams-Ardakani M. Mohagheghzadeh A. (2014). Paracelsus Experiments in a Persian Book on Compound Remedies, Amal-E-Saleh (1766 A.D.). Pharm. Hist. (Lond) 44, 48–51. PMID: 25029776. 25029776

[B16] Badr P. Sardari F. A. (2019). The Compote-like Nutraceutical of Naqoa: A Traditional Cholagogue Agent. Trad. Integr. Med. 4, 170–175. 10.18502/tim.v4i4.2137

[B17] Ballabh B. Chaurasia O. P. (2007). Traditional Medicinal Plants of Cold Desert Ladakh-Used in Treatment of Cold, Cough and Fever. J. Ethnopharmacology 112, 341–349. 10.1016/j.jep.2007.03.020 17459623

[B18] Barnes J. Anderson L. Phillipson J. (2013). Herbal Medicines. 4th edition. London: Pharmaceutical Press.

[B19] Barreca D. Nabavi S. M. Sureda A. Rasekhian M. Raciti R. Silva A. S. (2020). Almonds (*Prunus Dulcis* Mill. D. A. Webb): A Source of Nutrients and Health-Promoting Compounds. Nutrients 12, 672. 10.3390/nu12030672 PMC7146189 32121549

[B20] Bayarri S. Durán L. Costell E. (2004). Influence of Sweeteners on the Viscoelasticity of Hydrocolloids Gelled Systems. Food Hydrocolloids 18, 611–619. 10.1016/j.foodhyd.2003.10.004

[B21] Begum H. A. Asad F. Sadiq A. Mulk S. Ali K. (2019). Antioxidant, Antimicrobial Activity and Phytochemical Analysis of the Seeds Extract of Cucumis Sativus Linn. Pab 7, 433–441. 10.19045/bspab.2018.700202

[B22] Bekhit A. E.-D. A. Cheng V. J. McConnell M. Zhao J. H. Sedcole R. Harrison R. (2011). Antioxidant Activities, Sensory and Anti-influenza Activity of Grape Skin tea Infusion. Food Chem. 129, 837–845. 10.1016/j.foodchem.2011.05.032 25212307

[B23] Berman B. Ellis C. Elmets C. (2016). Polypodium Leucotomos—An Overview of Basic Investigative Findings. J. Drugs Dermatol. 15, 224–228. PMID: 26885792. 26885792 PMC5189711

[B24] Berretta A. A. Silveira M. A. D. Cóndor Capcha J. M. De Jong D. (2020). Propolis and its Potential against SARS-CoV-2 Infection Mechanisms and COVID-19 Disease. Biomed. Pharmacother. 131, 110622. 10.1016/j.biopha.2020.110622 32890967 PMC7430291

[B25] Bhakta T. Mukherjee P. K. Saha K. Pal M. Saha B. P. (1998). Studies on Antitussive Activity of *Cassia Fistula* (Leguminosae) Leaf Extract. Pharm. Biol. 36, 140–143. 10.1076/phbi.36.2.140.4598

[B26] Bhuiyan F. R. Howlader S. Raihan T. Hasan M. (2020). Plants Metabolites: Possibility of Natural Therapeutics against the Covid-19 Pandemic. Front. Med. 7, 444. 10.3389/fmed.2020.00444 PMC7427128 32850918

[B27] Boeriu C. G. Springer J. Kooy F. K. Van Den Broek L. A. M. Eggink G. (2013). Production Methods for Hyaluronan. Int. J. Carbohydr. Chem. 2013, 1–14. 10.1155/2013/624967

[B28] Bokov D. O. Sharipova R. I. Potanina O. G. Nikulin A. V. Nasser R. A. Samylina I. A. (2020). Polysaccharides of Crude Herbal Drugs as a Group of Biologically Active Compounds in the Field of Modern Pharmacognosy: Physicochemical Properties, Classification, Pharmacopoeial Analysis. Sys. Rev. Pharm. 11, 206–212. 10.31838/srp.2020.6.32

[B29] Bouaziz F. Koubaa M. Ellouz Ghorbel R. Ellouz Chaabouni S. (2017). Biological Properties of Water-Soluble Polysaccharides and Hemicelluloses from almond Gum. Int. J. Biol. Macromolecules 95, 667–674. 10.1016/j.ijbiomac.2016.11.104 27908717

[B30] Bouaziz F. Koubaa M. Ellouz Ghorbel R. Ellouz Chaabouni S. (2016). Recent Advances in Rosaceae Gum Exudates: From Synthesis to Food and Non-food Applications. Int. J. Biol. Macromolecules 86, 535–545. 10.1016/j.ijbiomac.2016.01.081 26836615

[B31] Bouaziz F. Koubaa M. Helbert C. B. Kallel F. Driss D. Kacem I. (2015). Purification, Structural Data and Biological Properties of Polysaccharide fromPrunus Amygdalusgum. Int. J. Food Sci. Technol. 50, 578–584. 10.1111/ijfs.12687

[B32] Boyd E. M. (1946). The Cough Syrup. Bmj 2, 735–736. 10.1136/bmj.2.4480.735 20273996 PMC2054729

[B33] Bozkurt H. Göğüş F. Eren S. (1999). Nonenzymic browning Reactions in Boiled Grape Juice and its Models during Storage. Food Chem. 64, 89–93. 10.1016/S0308-8146(98)00081-8

[B34] Brinckmann J. Sigwart H. van Houten Taylor L. (2003). Safety and Efficacy of a Traditional Herbal Medicine (Throat Coat) in Symptomatic Temporary Relief of Pain in Patients with Acute Pharyngitis: a Multicenter, Prospective, Randomized, Double-Blinded, Placebo-Controlled Study. J. Altern. Complement. Med. 9, 285–298. 10.1089/10755530360623400 12804082

[B35] Burlando B. Cornara L. (2013). Honey in Dermatology and Skin Care: a Review. J. Cosmet. Dermatol. 12, 306–313. 10.1111/jocd.12058 24305429

[B36] Butler L. M. Koh W.-P. Lee H.-P. Yu M. C. London S. J. (2004). Dietary Fiber and Reduced Cough with Phlegm. Am. J. Respir. Crit. Care Med. 170, 279–287. 10.1164/rccm.200306-789OC 15117740

[B37] Carfì A. Bernabei R. Landi F. (2020). Persistent Symptoms in Patients after Acute COVID-19. JAMA 324, 603–605. 10.1001/jama.2020.12603 32644129 PMC7349096

[B38] Cesarone M. R. Belcaro G. Hu S. Dugall M. Hosoi M. Ledda A. (2020). Supplementary Prevention and Management of Asthma with Quercetin Phytosome: a Pilot Registry. Minerva Med. 110, 524–529. 10.23736/S0026-4806.19.06319-5 31578841

[B39] Charles D. J. (2013). Antioxidant Roperties of Spices, Herbs and Other Sources. New York: Springer-Verlag. 10.1007/978-1-4614-4310-0

[B40] Chhillar H. Chopra P. Ashfaq M. A. (2020). Lignans from Linseed (Linum usitatissimumL.) and its Allied Species: Retrospect, Introspect and prospect. Crit. Rev. Food Sci. Nutr. 3, 1–23. 10.1080/10408398.2020.1784840 32619358

[B41] Chiru T. Fursenco C. Ciobanu N. Dinu M. Popescu E. Ancuceanu R. (2020). Use of Medicinal Plants in Complementary Treatment of the Common Cold and Influenza—Perception of Pharmacy Customers in Moldova and Romania. J. Herbal Med. 21, 100346. 10.1016/j.hermed.2020.100346

[B42] Chonpathompikunlert P. Boonruamkaew P. Sukketsiri W. Hutamekalin P. Sroyraya M. (2018). The Antioxidant and Neurochemical Activity of *Apium graveolens* L. And its Ameliorative Effect on MPTP-Induced Parkinson-like Symptoms in Mice. BMC. Complement. Altern. Med. 18, 103. 10.1186/s12906-018-2166-0 29558946 PMC5859653

[B43] Choudhry N. Zhao X. Xu D. Zanin M. Chen W. Yang Z. (2020). Chinese Therapeutic Strategy for Fighting COVID-19 and Potential Small-Molecule Inhibitors against Severe Acute Respiratory Syndrome Coronavirus 2 (SARS-CoV-2). J. Med. Chem. 63, 13205–13227. 10.1021/acs.jmedchem.0c00626 32845145

[B44] Clemency B. M. Varughese R. Scheafer D. K. Ludwig B. Welch J. V. McCormack R. F. (2020). Symptom Criteria for COVID‐19 Testing of Heath Care Workers. Acad. Emerg. Med. 27, 469–474. 10.1111/acem.14009 32396670 PMC7272901

[B45] Dai J. Wang G. Li W. Zhang L. Yang J. Zhao X. (2012). High-Throughput Screening for Anti-influenza A Virus Drugs and Study of the Mechanism of Procyanidin on Influenza A Virus-Induced Autophagy. J. Biomol. Screen. 17, 605–617. 10.1177/1087057111435236 22286278

[B46] Dehkhoda A. K. (1999). Dehkhoda Dictionary. Tehran: Tehran University Press.

[B47] Derosa G. Maffioli P. D'Angelo A. Di Pierro F. (2021). A Role for Quercetin in Coronavirus Disease 2019 (COVID‐19). Phytotherapy Res. 35, 1230–1236. 10.1002/ptr.6887 PMC7675685 33034398

[B48] Donma M. M. Donma O. (2020). The Effects of *Allium Sativum* on Immunity within the Scope of COVID-19 Infection. Med. Hypotheses 144, 109934. 10.1016/j.mehy.2020.109934 32512493 PMC7265825

[B49] Dotto J. M. Chacha J. S. (2020). The Potential of Pumpkin Seeds as a Functional Food Ingredient: A Review. Scientific Afr. 10, e00575. 10.1016/j.sciaf.2020.e00575

[B50] El-Saber Batiha G. Magdy Beshbishy A. G. Wasef L. Y. H. A. A. Al-Sagan A. Abd El-Hack M. E. Taha A. E. (2020). Chemical Constituents and Pharmacological Activities of Garlic (*Allium Sativum* L.): A Review. Nutrients 12, 872. 10.3390/nul2030872 PMC7146530 32213941

[B51] Esposito A. J. Bhatraju P. K. Stapleton R. D. Wurfel M. M. Mikacenic C. (2017). Hyaluronic Acid Is Associated with Organ Dysfunction in Acute Respiratory Distress Syndrome. Crit. Care 21, 304. 10.1186/s13054-017-1895-7 29237497 PMC5729515

[B52] Farjadmand F. Shams Ardekani M. R. Zargaran A. (2017). Wines as Pharmaceutical Dosage Forms in ‘Amal Saleh’, the Last Persian Pharmacopoeia in the Zand Era. Pharm. Hist. (Lond). 47, 8–10. 10.33029/9704-5345-2-gph-2020-1-96

[B53] Fattahi A. Petrini P. Munarin F. Shokoohinia Y. Golozar M. A. Varshosaz J. (2013). Polysaccharides Derived from Tragacanth as Biocompatible Polymers and Gels. J. Appl. Polym. Sci. 129, 2092–2102. 10.1002/app.38931

[B54] Fernández-López J. Botella-Martínez C. Navarro-Rodríguez de Vera C. Sayas-Barberá M. E. Viuda-Martos M. Sánchez-Zapata E. (2020). Vegetable Soups and Creams: Raw Materials, Processing, Health Benefits, and Innovation Trends. Plants 9, 1769. 10.3390/plants9121769 PMC7764940 33327480

[B55] Ferreira S. S. Passos C. P. Madureira P. Vilanova M. Coimbra M. A. (2015). Structure-function Relationships of Immunostimulatory Polysaccharides: a Review. Carbohydr. Polym. 132, 378–396. 10.1016/j.carbpol.2015.05.079 26256362

[B56] Fischer U. A. Carle R. Kammerer D. R. (2011). Identification and Quantification of Phenolic Compounds from Pomegranate (Punica Granatum L.) Peel, Mesocarp, Aril and Differently Produced Juices by HPLC-DAD-ESI/MSn. Food Chem. 127, 807–821. 10.1016/j.foodchem.2010.12.156 23140740

[B57] Footitt J. Johnston S. L. (2009). Cough and Viruses in Airways Disease: Mechanisms. Pulm. Pharmacol. Ther. 22, 108–113. 10.1016/j.pupt.2008.12.022 19480062 PMC7110775

[B58] François K. O. Balzarini J. (2012). Potential of Carbohydrate-Binding Agents as Therapeutics against Enveloped Viruses. Med. Res. Rev. 32, 349–387. 10.1002/med.20216 20577974 PMC7168447

[B59] Fraser E. (2020). Long Term Respiratory Complications of Covid-19. BMJ 370, m3001. 10.1136/bmj.m3001 32747332

[B60] Fu K.-L. Li X. Ye J. Lu L. Xu X.-K. Li H.-L. (2016). Chemical Constituents of *Narcissus Tazetta* Var. *Chinensis* and Their Antioxidant Activities. Fitoterapia 113, 110–116. 10.1016/j.fitote.2016.07.013 27476617

[B61] Fuzimoto A. D. Isidoro C. (2020). The Antiviral and Coronavirus-Host Protein Pathways Inhibiting Properties of Herbs and Natural Compounds—Additional Weapons in the Fight against the COVID-19 Pandemic?. J. Traditional Complement. Med. 10, 405–419. 10.1016/j.jtcme.2020.05.003 PMC7260130 32691005

[B62] Ganeshpurkar A. Saluja A. K. (2017). The Pharmacological Potential of Rutin. Saudi Pharm. J. 25, 149–164. 10.1016/j.jsps.2016.04.025 28344465 PMC5355559

[B63] Garvin M. R. Alvarez C. Miller J. I. Prates E. T. Walker A. M. Amos B. K. (2020). A Mechanistic Model and Therapeutic Interventions for COVID-19 Involving a RAS-Mediated Bradykinin Storm. eLife 9, e59177. 10.7554/eLife.59177 32633718 PMC7410499

[B64] Georgiev V. Ananga A. Tsolova V. (2014). Recent Advances and Uses of Grape Flavonoids as Nutraceuticals. Nutrients 6, 391–415. 10.3390/nu6010391 24451310 PMC3916869

[B65] Ghaeni Heravi M. S. (1766). Qarabadin-e-Salehi. Rewritten by Badr P, Mohagheghzadeh A, Shams Ardakani MR. 1st ed. Tehran: Choogan.

[B66] Ghahraman A. Okhovvat A. R. (2004). Matching the Old Medicinal Plant Names with Scientific Terminology. Tehran: Tehran University Press.

[B67] Gharashi A. (1288). Alshamel-fi Alsanaat Altebya. Tehran: Iran University of Medical Sciences Publication.

[B68] Gharibzahedi S. M. T. (2018). Favorite and Traditional rice Flour-Based Puddings, Breads, and Pastries in the north of Iran: A Review. J. Ethnic Foods 5, 105–113. 10.1016/j.jef.2018.03.001

[B69] Giannoni E. Baud D. Agri V. D. Gibson G. R. Reid G. (2020). Probiotics and COVID-19. Lancet Gastroenterol. Hepatol. 5, 720–721. 10.1016/s2468-1253(20)30195-3 PMC7357984 32673603

[B70] Gibson G. R. Roberfroid M. B. (1995). Dietary Modulation of the Human Colonic Microbiota: Introducing the Concept of Prebiotics. J. Nutr. 125, 1401–1412. 10.1093/jn/125.6.1401 7782892

[B71] Giordo R. Zinellu A. Eid A. H. Pintus G. (2021). Therapeutic Potential of Resveratrol in COVID-19-Associated Hemostatic Disorders. Molecules 26, 856. 10.3390/molecules26040856 33562030 PMC7915700

[B72] Gordon C. J. Tchesnokov E. P. Woolner E. Perry J. K. Feng J. Y. Porter D. P. (2020). Remdesivir Is a Direct-Acting Antiviral that Inhibits RNA-dependent RNA Polymerase from Severe Acute Respiratory Syndrome Coronavirus 2 with High Potency. J. Biol. Chem. 295, 6785–6797. 10.1074/jbc.RA120.013679 32284326 PMC7242698

[B73] Gu Y.-Y. Zhang M. Cen H. Wu Y.-F. Lu Z. Lu F. (2021). Quercetin as a Potential Treatment for COVID-19-Induced Acute Kidney Injury: Based on Network Pharmacology and Molecular Docking Study. PLoS One 16, e0245209. 10.1371/journal.pone.0245209 33444408 PMC7808608

[B74] Haas I. C. S. Toaldo I. S. Gomes T. M. Luna A. S. Gois J. S. Bordignon-Luiz M. T. (2018). Polyphenolic Profile, Macro- and Microelements in Bioaccessible Fractions of Grape Juice Sediment Using *In Vitro* Gastrointestinal Simulation. Food Biosci. 27, 66–74. 10.1016/j.fbio.2018.11.002

[B75] Hafsa J. Chaouch M. A. CharfeddineRihouey B. C. Rihouey C. Limem K. Le Cerf D. (2017). Effect of Ultrasonic Degradation of Hyaluronic Acid Extracted from Rooster Comb on Antioxidant and Antiglycation Activities. Pharm. Biol. 55, 156–163. 10.1080/13880209.2016.1232740 27650976 PMC7011968

[B76] Hamedi A. Zarshenas M. M. Sohrabpour M. Zargaran A. (2013). Herbal Medicinal Oils in Traditional Persian Medicine. Pharm. Biol. 51, 1208–1218. 10.3109/13880209.2013.777462 23746335

[B77] He B. Garmire L. (2020). Prediction of Repurposed Drugs for Treating Lung Injury in COVID-19. F1000Res 9, 609. 10.12688/f1000research.23996.2 32934806 PMC7468567

[B78] He Y.-Q. Zhou C.-C. Yu L.-Y. Wang L. Deng J.-l. Tao Y.-L. (2021). Natural Product Derived Phytochemicals in Managing Acute Lung Injury by Multiple Mechanisms. Pharmacol. Res. 163, 105224. 10.1016/j.phrs.2020.105224 33007416 PMC7522693

[B79] Huang W. Wen Z. Wang M. Xu B. Zhou B. Li X. (2020b). Anticomplement and Antitussive Activities of Major Compound Extracted from Chimonanthus Nitens Oliv. Leaf. Biomed. Chromatogr. 34, e4736. 10.1002/bmc.4736 31696526

[B80] Huang W. Wen Z. Wu H. Wang Q. Zhou Li., Y. (2020a). Primary Research on Components with Anticomplement and Antitussive Activities from Leave Extracts of Chimonanthus Nitens. Chin. Trad. Herb. Drugs 51, 3869–3875.

[B81] Ibrahim S. R. M. Khedr A. I. M. Mohamed G. A. Zayed M. F. El-Kholy A. A.-E. S. Al Haidari R. A. (2019). Cucumol B, a New Triterpene Benzoate from *Cucumis Melo* Seeds with Cytotoxic Effect toward Ovarian and Human Breast Adenocarcinoma. J. Asian Nat. Prod. Res. 21, 1112–1118. 10.1080/10286020.2018.1488832 29947257

[B82] Irwin R. S. Dudiki N. French C. L. Abu Dabrh A. M. Altman K. W. Azoulay E. (2020). Life-Threatening and Non-life-threatening Complications Associated with Coughing. Chest 158, 2058–2073. 10.1016/j.chest.2020.06.012 32565267 PMC8640838

[B83] Isfahlan A. J. Mahmoodzadeh A. Hassanzadeh A. Heidari R. Jamei R. (2010). Antioxidant and Antiradical Activities of Phenolic Extracts from Iranian almond (*Prunus Amygdalus* L.) Hulls and Shells. Turk. J. Biol. 34, 165–173. 10.3906/biy-0807-21

[B84] Iwu M. Okunji C. Tchimene M. Anele N. Chah K. Osonwa U. (2009). Stability of Cough Linctus (Streptol) Formulated from Named Medicinal Plant Extracts. Chem. Pharm. Bull. 57, 229–232. 10.1248/cpb.57.229 19252311

[B85] Jackson W. A. (2001). A Short Guide to Humoral Medicine. Trends Pharmacol. Sci. 22, 487–489. 10.1016/s0165-6147(00)01804-6 11543877

[B86] Jafari F. Khodaiyan F. Kiani H. Hosseini S. S. (2017). Pectin from Carrot Pomace: Optimization of Extraction and Physicochemical Properties. Carbohydr. Polym. 157, 1315–1322. 10.1016/j.carbpol.2016.11.013 27987838

[B87] Jalali A. Dabaghian F. Akbrialiabad H. Foroughinia F. Zarshenas M. M. (2020). A Pharmacology‐based Comprehensive Review on Medicinal Plants and Phytoactive Constituents Possibly Effective in the Management of COVID ‐19. Phytotherapy Res. 35, 1925–1938. 10.1002/ptr.6936 33159391

[B88] Jang B. C. Sim H. S. Jeong B. Y. Park H. M. Oh M. J. (2008). Isolation of Cucurbitacin E from Pumpkin Seed and Analysis of its Anti-cancer and Anti-inflammatory Activities. FASEB J. 22, 889. 10.1096/fasebj.22.1_supplement.889.6

[B89] Jeong C.-S. (2009). Evaluation for Protective Effect of Rutin, a Natural Flavonoid, against HCl/ethanol-Induced Gastric Lesions. Biomolecules Ther. 17, 199–204. 10.4062/biomolther.2009.17.2.199

[B90] Johnson P. Arif A. A. Lee-Sayer S. S. M. Dong Y. (2018). Hyaluronan and its Interactions with Immune Cells in the Healthy and Inflamed Lung. Front. Immunol. 9, 2787. 10.3389/fimmu.2018.02787 30555472 PMC6281886

[B91] Kaithwas G. Majumdar D. K. (2012). *In Vitro* antioxidant and *In Vivo* Antidiabetic, Antihyperlipidemic Activity of Linseed Oil against Streptozotocin-Induced Toxicity in Albino Rats. Eur. J. Lipid Sci. Technol. 114, 1237–1245. 10.1002/ejlt.201100263

[B92] Kardošová A. Machová E. (2006). Antioxidant Activity of Medicinal Plant Polysaccharides. Fitoterapia 77, 367–373. 10.1016/j.fitote.2006.05.001 16797146

[B93] Kaveh M. Eftekhar N. Boskabady M. H. (2019). The Effect of Alpha Linolenic Acid on Tracheal Responsiveness, Lung Inflammation, and Immune Markers in Sensitized Rats. Iran J. Basic Med. Sci. 22, 255–261. 10.22038/ijbms.2019.27381.6684 31156785 PMC6528709

[B94] Kelm G. R. Wickett R. R. (2017). The Role of Fatty Acids in Cosmetic Technology. Fatty Acids 17, 385–404. 10.1016/b978-0-12-809521-8.00012-x

[B95] Khalifa S. A. M. Yosri N. El-Mallah M. F. Ghonaim R. Guo Z. Musharraf S. G. (2021). Screening for Natural and Derived Bio-Active Compounds in Preclinical and Clinical Studies: One of the Frontlines of Fighting the Coronaviruses Pandemic. Phytomedicine 85, 153311. 10.1016/j.phymed.2020.153311 33067112 PMC7455571

[B96] Khalil M. Salih M. Mustafa A. (2020). Broad Beans (Vicia faba) and the Potential to Protect from COVID-19 Coronavirus Infection. Sudan J. Paed 20, 10–12. 10.24911/sjp.1061585398078 PMC7282436 32528195

[B97] Khanna K. Kohli S. K. Kaur R. Bhardwaj A. Bhardwaj V. Ohri P. (2021). Herbal Immune-Boosters: Substantial Warriors of Pandemic Covid-19 Battle. Phytomedicine 85, 153361. 10.1016/j.phymed.2020.153361 33485605 PMC7532351

[B98] Kheterpal K. Khanna T. Arora R. B. (1989). *In Vitro* and *In Vivo* Bronchorelaxant Effect in guinea Pigs of “joshina”—A Herbal Polypharmaceutical. J. Ethnopharmacology 26, 183–187. 10.1016/0378-8741(89)90065-2 2601358

[B203] Kirkpatrick G. L (1996). The Common Cold. Prim Care 23, 657–675. 10.1016/s0095-4543(05)70355-9 8890137 PMC7125839

[B99] Kooti W. Daraei N. (2017). A Review of the Antioxidant Activity of Celery (*Apium graveolens* L). J. Evid. Based. Complement. Altern. Med. 22, 1029–1034. 10.1177/2156587217717415 PMC5871295 28701046

[B100] Korman A. M. Reynolds K. A. Nabhan F. Konda B. Shah M. H. Kaffenberger B. H. (2019). Vandetanib-induced Phototoxic Drug Eruption Treated with *Polypodium Leucotomos* Extract: A Case Report and Review of the Literature. J. Clin. Aesthet. Dermatol. 12, 35–38. 10.32388/tisq3c PMC6937146 32038747

[B101] Kornienko A. Evidente A. (2008). Chemistry, Biology, and Medicinal Potential of Narciclasine and its Congeners. Chem. Rev. 108, 1982–2014. 10.1021/cr078198u 18489166 PMC2856661

[B102] Kostadinović Veličkovska S. Catalin Moţ A. Mitrev S. Gulaboski R. Brühl L. Mirhosseini H. (2018). Bioactive Compounds and “*In Vitro*” Antioxidant Activity of Some Traditional and Non-traditional Cold-Pressed Edible Oils from Macedonia. J. Food Sci. Technol. 55, 1614–1623. 10.1007/s13197-018-3050-0 29666513 PMC5897278

[B103] Krystallis A. Maglaras G. Mamalis S. (2008). Motivations and Cognitive Structures of Consumers in Their Purchasing of Functional Foods. Food Qual. Preference 19, 525–538. 10.1016/j.foodqual.2007.12.005

[B104] Lansky E. P. Newman R. A. (2007). *Punica Granatum* (Pomegranate) and its Potential for Prevention and Treatment of Inflammation and Cancer. J. Ethnopharmacology 109, 177–206. 10.1016/j.jep.2006.09.006 17157465

[B105] Lazarus J. V. Ratzan S. C. Palayew A. Gostin L. O. Larson H. J. Rabin K. (2021). A Global Survey of Potential Acceptance of a COVID-19 Vaccine. Nat. Med. 27, 225–228. 10.1038/s41591-020-1124-9 33082575 PMC7573523

[B106] Leung P. C. (2015). “Use of Animal Fats in Traditional Chinese Medicine,” in Regenerative Medicine. Editors Bhattacharya N. Stubblefield P. (London: Springer), 73–76. 10.1007/978-1-4471-6542-2_8

[B107] Li M.-Y. Feng K. Hou X.-L. Jiang Q. Xu Z.-S. Wang G.-L. (2020). The Genome Sequence of Celery (*Apium graveolens* L.), an Important Leaf Vegetable Crop Rich in Apigenin in the Apiaceae Family. Hortic. Res. 7, 9. 10.1038/s41438-019-0235-2 31934340 PMC6944684

[B108] Li R. Wu K. Li Y. Liang X. Tse W. K. F. Yang L. (2020). Revealing the Targets and Mechanisms of Vitamin A in the Treatment of COVID-19. Aging 12, 15784–15796. 10.18632/aging.103888 32805728 PMC7467385

[B109] Li X. Ma X. (2020). Acute Respiratory Failure in COVID-19: Is it “typical” ARDS?. Crit. Care 24, 198. 10.1186/s13054-020-02911-9 32375845 PMC7202792

[B110] Lima W. G. Brito J. C. M. Cruz Nizer W. S. (2020). Bee Products as a Source of Promising Therapeutic and Chemoprophylaxis Strategies against COVID ‐19 ( SARS‐CoV ‐2). Phytotherapy Res. 35, 743–750. 10.1002/ptr.6872 PMC7536959 32945590

[B111] Lu Y. Yu T. Liu J. Gu L. (2018). Vitexin Attenuates Lipopolysaccharide-Induced Acute Lung Injury by Controlling the Nrf2 Pathway. PLoS One 13, e0196405. 10.1371/journal.pone.0196405 29694408 PMC5942793

[B112] Mahboubi M. (2020). Marsh Mallow (*Althaea Officinalis* L.) and its Potency in the Treatment of Cough. Complement. Med. Res. 27, 174–183. 10.1159/000503747 31770755

[B113] Majumder R. Mandal M. (2020). Screening of Plant-Based Natural Compounds as a Potential COVID-19 Main Protease Inhibitor: An In Silico Docking and Molecular Dynamics Simulation Approach. J. Biomol. Struct. Dyn. 14, 1–16. 10.1080/07391102.2020.1817787 PMC7544942 32897138

[B114] Maroli N. Bhasuran B. Natarajan J. Kolandaivel P. (2020). The potential role of procyanidin as a therapeutic agent against SARS-CoV-2: A text mining, molecular docking and molecular dynamics simulation approach. J. Biomol. Struct. Dyn. 12, 1–16. 10.1080/07391102.2020.1823887 PMC7544928 32960159

[B115] Martinez M. A. (2020). Clinical Trials of Repurposed Antivirals for SARS-CoV-2. Antimicrob. Agents Chemother. 64, e01101–20. 10.1128/AAC.01101-20 32631826 PMC7449177

[B116] Marze S. (2017). Bioavailability of Nutrients and Micronutrients: Advances in Modeling and *In Vitro* Approaches. Annu. Rev. Food Sci. Technol. 8, 35–55. 10.1146/annurev-food-030216-030055 28068491

[B117] Matteo G. D. Spano M. Grosso M. Salvo A. Ingallina C. Russo M. (2020). Food and COVID-19: Preventive/Co-Therapeutic Strategies Explored by Current Clinical Trials and *In Silico* Studies. Foods 9, 1036. 10.3390/foods9081036 PMC7466271 32752217

[B118] Montesano D. Rocchetti G. Putnik P. Lucini L. (2018). Bioactive Profile of Pumpkin: an Overview on Terpenoids and Their Health-Promoting Properties. Curr. Opin. 22, 81–87. 10.1016/j.cofs.2018.02.003

[B119] Mousavi S. H. Naghizade B. Pourgonabadi S. Ghorbani A. (2016). Protective Effect of *Viola Tricolor* and *Viola Odorata* Extracts on Serum/glucose Deprivation-Induced Neurotoxicity: Role of Reactive Oxygen Species. Avicenna J. Phytomed. 6, 434–441. 10.32388/tisq3c 27516984 PMC4967839

[B120] Muhammad N. Saeed M. Khan H. (2012). Antipyretic, Analgesic and Anti-inflammatory Activity of *Viola Betonicifolia* Whole Plant. BMC. Complement. Altern. Med. 12, 59. 10.1186/1472-6882-12-59 22551220 PMC3419074

[B121] Mulholland S. Chang A. B. (2009). Honey and Lozenges for Children with Nonspecific Cough. Cochrane Database Syst. Rev. 2009, CD007523. 10.1002/14651858.CD007523.pub2 PMC7202236 19370690

[B122] Naidu M. M. Vedashree M. Satapathy P. Khanum H. Ramsamy R. Hebbar H. U. (2016). Effect of Drying Methods on the Quality Characteristics of Dill (*Anethum Graveolens*) Greens. Food Chem. 192, 849–856. 10.1016/j.foodchem.2015.07.076 26304420

[B123] Naik S. R. Bharadwaj P. Dingelstad N. Kalyaanamoorthy S. Mandal S. C. Ganesan A. (2021). Structure-based Virtual Screening, Molecular Dynamics and Binding Affinity Calculations of Some Potential Phytocompounds against SARS-CoV-2. J. Biomol. Struct. Dyn. 8, 1–18. 10.1080/07391102.2021.1891969 33682632

[B124] Navajas-Porras B. Pérez-Burillo S. Valverde-Moya Á. J. Hinojosa-Nogueira D. Pastoriza S. Rufián-Henares J. Á. (2020). Effect of Cooking Methods on the Antioxidant Capacity of Plant Foods Submitted to *In Vitro* Digestion-Fermentation. Antioxidants (Basel). 9, 1312. 10.3390/antiox9121312 PMC7767424 33371445

[B125] Nayak B. Liu R. H. Tang J. (2015). Effect of Processing on Phenolic Antioxidants of Fruits, Vegetables, and Grains-Aa Review. Crit. Rev. Food Sci. Nutr. 55, 887–919. 10.1080/10408398.2011.654142 24915381

[B126] Nayeb morad F. Rashidi A. Khajavi R. Rahimi M. Bahador A. (2018). Production of Wound Dressing with Nano Fibers Contain Bassorin/Ofloxacin for Improvement Burn Wound. Nanomed. Res. J. 3, 180–189. 10.22034/nmrj.2018.04.002

[B127] Nikaein F. Zargaran A. Mehdizadeh A. (2012). Rhazes’ Concepts and Manuscripts on Nutrition in Treatment and Health Care. Anc. Sci. Life 31, 160–163. 10.4103/0257-7941.107357 23661862 PMC3644752

[B128] Nikhat S. Fazil M. (2020). Overview of Covid-19: Its Prevention and Management in the Light of Unani Medicine. Sci. Total Environ. 728, 138859. 10.1016/j.scitotenv.2020.138859 32334163 PMC7174982

[B129] Noble P. W. Liang J. Jiang D. (2011). Hyaluronan as an Immune Regulator in Human Diseases. Physiol. Rev. 91, 221–264. 10.1152/physrev.00052.2009 21248167 PMC3051404

[B130] Noreen H. Madeeha R. Zarmina R. K. Minhas A. Jan M. Hassan W. (2019). Biochemical Analysis and mineral Composition of Methanolic Extract of *Astragalus gummifer* . Biomed. J. Sci. Tech. Res. 20, 14736–14741. 10.26717/BJSTR.2019.21.003609

[B131] Nosál'ova G. Strapková A. Kardosová A. Capek P. Zathurecký L. Bukovská E. (1992). Antitussive Action of Extracts and Polysaccharides of Marsh Mallow (Althea Officinalis L., Var. Robusta). Pharmazie 47, 224–226. 1615030

[B132] Nosalova G. Jurecek L. Hromadkova Z. Kostalova Z. Sadlonova V. (2013). Antioxidant Activity of Herbal Polysaccharides and Cough Reflex. Adv. Exp. Med. Biol. 788, 51–57. 10.1007/978-94-007-6627-3_8 23835958

[B133] Nosalova G. Mokry J. Franova S. (2006). Pharmacological Modulation of Cough Reflex. Adv. Phytomed. 2, 87–110. 10.1016/S1572-557X(05)02006-4

[B134] Nosáľová G. Prisenžňáková L. Košťálová Z. Ebringerová A. Hromádková Z. (2011). Suppressive Effect of Pectic Polysaccharides from *Cucurbita Pepo* L. Var. *Styriaca* on Citric Acid-Induced Cough Reflex in guinea Pigs. Fitoterapia 82, 357–364. 10.1016/j.fitote.2010.11.006 21062638

[B135] Oduwole O. Udoh E. E. Oyo-Ita A. Meremikwu M. M. (2018). Honey for Acute Cough in Children (Review). Cochrane Database Syst. Rev. 4, CD007094. 10.1002/14651858.CD007094.pub5 29633783 PMC6513626

[B136] Oladele J. O. Ajayi E. I. Oyeleke O. M. Oladele O. T. Olowookere B. D. Adeniyi B. M. (2020). A Systematic Review on COVID-19 Pandemic with Special Emphasis on Curative Potentials of Nigeria Based Medicinal Plants. Heliyon 6, e04897. 10.1016/j.heliyon.2020.e04897 32929412 PMC7480258

[B137] Oliveira T. T. Campos K. M. Cerqueira-Lima A. T. da Silva Velozo E. Ribeiro Melo I. C. (2015). Cana Brasil CarneiroPotential Therapeutic Effect of *Allium cepa* L. And Quercetin in a Murine Model of *Blomia Tropicalis* Induced Asthma. Daru 23, 18. 10.1186/s40199-015-0098-5 25890178 PMC4344790

[B138] Ooi L. S. M. Ho W. S. Ngai K. L. K. TianChan L. P. K. S. Sun S. S. M. (2010). *Narcissus Tazetta* Lectin Shows strong Inhibitory Effects against Respiratory Syncytial Virus, Influenza A (H1N1, H3N2, H5N1) and B Viruses. J. Biosci. 35, 95–103. 10.1007/s12038-010-0012-8 20413914 PMC7091448

[B139] Oshaghi E. A. Khodadadi I. Tavilani H. Goodarzi M. T. (2016). Aqueous Extract of *Anethum Graveolens* L. Has Potential Antioxidant and Antiglycation Effects. Iran J. Med. Sci. 41, 328–333. 27365555 PMC4912652

[B140] Papu S. Jaivir S. Sweta S. Singh B. R. (2014). Medicinal Values of Garlic (*Allium Sativum* L.) in Human Life: An Overview. Greener J. Agric. Sci. 4, 265–280. 10.15580/GJAS.2014.6.031914151

[B141] Parada J. Aguilera J. M. (2007). Food Microstructure Affects the Bioavailability of Several Nutrients. J. Food Sci. 72, R21–R32. 10.1111/j.1750-3841.2007.00274.x 17995848

[B142] Park J. M. Han Y. M. Kangwan N. Lee S. Y. Jung M. K. Kim E. H. (2014). S-allyl Cysteine Alleviates Nonsteroidal Anti-inflammatory Drug-Induced Gastric Mucosal Damages by Increasing Cyclooxygenase-2 Inhibition, Heme Oxygenase-1 Induction, and Histone Deacetylation Inhibition. J. Gastroenterol. Hepatol. 29, 80–92. 10.1111/jgh.12730 25521739

[B143] Pasricha V. Satpathy G. Gupta R. K. (2014). Phytochemical and Antioxidant Activity of Underutilized Legume *Vicia faba* Seeds and Formulation of its Fortified Biscuits. J. Pharmacogn. Phytochem. 3, 75–80.

[B144] Pawar A. V. Killedar S. G. (2017). Uses of *Cassia Fistula* L. As a Medicinal Plant. Int. J. Adv. Res. Dev. 2, 85–91.

[B145] Peter K. V. (2012). Handbook of Herbs and Spices. Philadelphia: Woodhead Publishing.

[B146] Prabhu S. D. Rajeswari D. V. (2018). Nutritional and Biological Properties of *Vicia faba* L.: A Perspective Review. Int. Food Res. J. 25, 1332–1340.

[B147] Qasemzadeh M. J. Sharifi H. Hamedanian M. Gharehbeglou M. Heydari M. Sardari M. (2015). The Effect of *Viola Odorata* Flower Syrup on the Cough of Children with Asthma: A Double-Blind, Randomized Controlled Trial. Evid. Based. Complement. Altern. Med. 20, 287–291. 10.1177/2156587215584862 25954025

[B148] Qi J. Hu-Hu W. Zhang W. W. Deng S. L. Zhou G. H. Xu X. L. (2018). Identification and Characterization of the Proteins in Broth of Stewed Traditional Chinese Yellow-Feathered Chickens. Poult. Sci. 97, 1852–1860. 10.3382/ps/pey003 29462461

[B149] Qi X. Al‐Ghazzewi F. Tester R. (2018). Flour—Cooked or Uncooked?: A Healthy Food Component. Starch 70, 1700343. 10.1002/star.201700343

[B150] Ragone M. I. Sella M. Conforti P. Volonté M. G. Consolini A. E. (2007). The Spasmolytic Effect of *Aloysia Citriodora*, Palau (South American Cedrón) Is Partially Due to its Vitexin but Not Isovitexin on Rat Duodenums. J. Ethnopharmacol. 113, 258–266. 10.1016/j.jep.2007.06.003 17640836

[B151] Rahim H. Sadiq A. Khan S. Khan M. A. Amin F. Jan N. U. (2018). *Prunus Armeniaca* and *Prunus Domestica* Gums: Exploring Their Synergistic Binding Potential in Tablets. Lat. Am. J. Pharm. 37, 1672–1683.

[B152] Ramdani L. H. Bachari K. (2020). Potential Therapeutic Effects of Resveratrol against SARS-CoV-2. Acta Virol. 64, 276–280. 10.4149/av_2020_309 32985211

[B153] Reddy M. K. Gupta S. K. Jacob M. R. Khan S. I. Ferreira D. (2007). Antioxidant, Antimalarial and Antimicrobial Activities of Tannin-Rich Fractions, Ellagitannins and Phenolic Acids from *Punica Granatum* L. Planta Med. 53, 461–467. 10.1055/s-2007-967167 17566148

[B154] Roberfroid M. B. (2002). Global View on Functional Foods: European Perspectives. Br. J. Nutr. 88, 133–138. 10.1079/BJN2002677 12495454

[B155] Saadat S. Shakeri F. Boskabady M. H. (2018). Comparative Antitussive Effects of Medicinal Plants and Their Constituents. Altern. Therap. Health Med. 24, 36–49. 29332022

[B156] Sánchez-Rodríguez C. Cruces K. R. P. Riescoc L. R. García-Vela J. A. Sanz-Fernándeze R. (2018). Immunomodulatory Effect of *Polypodium Leucotomos* (Anapsos) in Child palatine Tonsil Model. Int. J. Pediatr. Otorhinolaryngol. 107, 56–61. 10.1016/j.ijporl.2018.01.030 29501312

[B157] Sant’Ana H. M. P. Stringheta P. C. Brandão S. C. C. Azeredo R. M. C. (1998). Carotenoid Retention and Vitamin A Value in Carrot (*Daucus Carota* L.) Prepared by Food Service. Food Chem. 61, 145–151. 10.1016/S0308-8146(97)00084-8

[B158] Santos M. A. Franco F. N. Caldeira C. A. de Araújo G. R. Vieira A. Chaves M. M. (2021). Antioxidant Effect of Resveratrol: Change in MAPK Cell Signaling Pathway during the Aging Process. Arch. Gerontol. Geriatr. 92, 104266. 10.1016/j.archger.2020.104266 33070070

[B159] Schnorr S. L. Crittenden A. N. Henry A. G. (2016). Impact of Brief Roasting on Starch Gelatinization in Whole Foods and Implications for Plant Food Nutritional Ecology in Human Evolution. Ethnoarchaeology 8, 30–56. 10.1080/19442890.2016.1150629

[B160] Seliya A. R. Patel N. K. (2009). Ethnomedicinal Uses of Climbers from Saraswati River Region of Patan District, North Gujarat. Ethnobotanical Leaflets 2009, 5. 10.21474/ijar01/11777

[B161] Shafiee M. Heidari A. Amouzegar H. Khani S. Nojavan F. (2019). Evaluation of the Effect of Roasted Lentil Flour (Lentil Saviq) as a Functional Food in Menstrual Bleeding Reduction. Complement. Ther. Med. 44, 27–31. 10.1016/J.CTIM.2019.03.010 31126566

[B162] Shawan M. M. A. K. Halder S. K. Hasan M. A. (2021). Luteolin and Abyssinone II as Potential Inhibitors of SARS-CoV-2: an In Silico Molecular Modeling Approach in Battling the COVID-19 Outbreak. Bull. Natl. Res. Cent. 45, 27. 10.1186/s42269-020-00479-6 33495684 PMC7816153

[B163] Shekh S. Reddy K. K. A. Gowd K. H. (2020). *In Silico* allicin Induced *S*-Thioallylation of SARS-CoV-2 Main Protease. J. Sulphur Chem. 42, 1–12. 10.1080/17415993.2020.1817457

[B164] Shekhar H. U. Howlader Z. H. Kabir Y. (2017). Exploring the Nutrition and Health Benefits of Functional Foods. Hershey, PA: IGI Global. 10.4018/978-1-5225-0591-4

[B165] Simona D. M. Carmen F. Franco Z. Maria I. (2009). Phenolic Glycosides from *Cucumis Melo* Var. *Inodorous* Seeds. Phytochem. Lett. 2, 130–133. 10.1016/j.phytol.2009.04.001

[B166] Singh A. Mishra A. (2020). Leucoefdin a Potential Inhibitor against SARS CoV-2 Mpro. J. Biomol. Struct. Dyn. 2020, 1–6. 10.1080/07391102.2020.1777903 PMC7309301 34281489

[B167] Singh V. K. Singh D. K. (2008). Pharmacological Effects of Garlic (*Allium Sativum* L.). Annu. Rev. Biomed. Sci. 1, 10. 10.5016/1806-87742008.v10p6

[B168] Sirasi N. (1990). Medieval and Early Renaissance Medicine: An Introduction to Knowledge and Practice. Chicago: University of Chicago Press.

[B169] Soleymani S. Zargaran A. (2018). From Food to Drug: Avicenna’s Perspective, a Brief Review. Res. J. Pharmacogn. 5, 65–69. 10.22127/RJP.2018.58509

[B170] Solivellas B. M. Martin T. C. (2012). *Polypodium Leucotomos* Extract Use to Prevent and Reduce the Risk of Infectious Diseases in High Performance Athletes. Infect. Drug Resist. 5, 149–153. 10.2147/IDR.S29113 23093910 PMC3476750

[B171] Solnier J. Fladerer J. P. (2020). Flavonoids: A Complementary Approach to Conventional Therapy of COVID-19?. Phytochem. Rev. 18, 1–23. 10.1007/s11101-020-09720-6 PMC7500502 32982616

[B172] Sriwijitalai W. Wiwanitkit V. (2020). Herbs that Might Be Effective for the Management of COVID-19: A Bioinformatics Analysis on Anti-tyrosine Kinase Property. J. Res. Med. Sci. 25, 44. 10.4103/jrms.JRMS_312_20 32582350 PMC7306235

[B173] Stephen A. M. Phillips G. O. (2006). Food Polysaccharides and Their Applications (Food Science and Technology**)**. Boca Raton, Florida: CRC Press.

[B174] Struyf T. Deeks J. J. Dinnes J. Takwoingi Y. Davenport C. Leeflang M. M. G. (2020). Signs and Symptoms to Determine if a Patient Presenting in Primary Care or Hospital Outpatient Settings Has COVID-19 Disease. Cochrane Database Syst. Rev. 7, CD013665. 10.1002/14651858.CD013665 32633856 PMC7386785

[B175] Subbarao K. Mahanty S. (2020). Respiratory Virus Infections: Understanding COVID-19. Immunity 52, 905–909. 10.1016/j.immuni.2020.05.004 32497522 PMC7237932

[B176] Subhashinee S. K. Wijeatne S. S. K. Abou-Zaid M. M. Shahidi F. (2006). Antioxidant Polyphenols in Almond and its Coproducts. J. Agric. Food Chem. 54, 312–318. 10.1021/jf051692j 16417285

[B177] Swamy M. K. (2020). Plant-derived Bioactives. Production, Properties and Therapeutic Applications. Singapore: Springer.

[B178] Swann D. A. (1968). Studies on Hyaluronic Acid. I. The Preparation and Properties of Rooster Comb Hyaluronic Acid. Biochim. Biophys. Acta 156, 17–30. 10.1016/0304-4165(68)90099-8 5645738

[B179] Syed D. N. Chamcheu J. C. Adhami V. M. Mukhtar H. (2013). Pomegranate Extracts and Cancer Prevention: Molecular and Cellular Activities. Anticancer Agents Med. Chem. 13, 1149–1161. 10.2174/1871520611313080003 23094914 PMC4052369

[B180] Talebi M. Talebi M. Farkhondeh T. Samarghandian S. (2020). Molecular Mechanism-Based Therapeutic Properties of Honey. Biomed. Pharmacother. 130, 1105902. 10.1016/j.biopha.2020.110590 32768885

[B181] Tanveer S. A. Latif A. B. Ashiq K. A. Qayyum M. E. Bajwa M. A. (2019). A Comprehensive Review on Pharmacological and Phytochemical Potential of *Cassia Fistula* L.: A Magical Herb. Int. J. Biol. Pharm. Allied Sci. 8, 1134–1157. 10.31032/ijbpas/2019/8.6.4734

[B182] Thakur N. Raigond P. Singh Y. Mishra T. Singh B. Lal M. K. (2020). Recent Updates on Bioaccessibility of Phytonutrients. Trends Food Sci. Technol. 97, 366–380. 10.1016/j.tifs.2020.01.019

[B183] The Plant List. 2013. Version 1.1. http://www.theplantlist.org/[Accessed October 30, 2020].

[B184] Thota S. M. Balan V. Sivaramakrishnan V. (2020). Natural Products as home-based Prophylactic and Symptom Management Agents in the Setting of COVID-19. Phytother Res. 34 (12), 3148–3167. 10.1002/ptr.6794 32881214 PMC7461159

[B185] Thuy B. T. P. My T. T. A. Hai N. T. T. Hieu L. T. Hoa T. T. Thi Phuong Loan H. (2020). Investigation into SARS-CoV-2 Resistance of Compounds in Garlic Essential Oil. ACS. Omega. 5, 8312–8320. 10.1021/acsomega.0c00772 32363255 PMC7123907

[B186] Tian C. Liu X. Chang Y. Wang R. Lv T. Cui C. (2021). Investigation of the Anti-inflammatory and Antioxidant Activities of Luteolin, Kaempferol, Apigenin and Quercetin. S. Afr. J. Bot. 137, 257–264. 10.1016/j.sajb.2020.10.022

[B187] Tobyn G. Denham A. Whitelegg M. (2011). The Western Herbal Tradition: 2000 Years of Medicinal Plant Knowledge. Philadelphia: Churchill Living Stone Elsevier, 337–348.

[B188] Torkova A. A. Lisitskaya K. V. Filimonov I. S. Glazunova O. A. KachalovaGolubev G. S. V. N. Fedorova T. V. (2018). Physicochemical and Functional Properties of *Cucurbita Maxima* Pumpkin Pectin and Commercial Citrus and Apple Pectins: A Comparative Evaluation. Plos. One. 13, e0204261. 10.1371/journal.pone.0204261 30235297 PMC6147495

[B189] Uden W. Pras N. Woerdenbag H. J. (1994). “ *Linum* Species (Flax): *In Vivo* and *In Vitro* Accumulation of Lignans and Other Metabolites,” in Medicinal and Aromatic Plants VI. Biotechnology in Agriculture and Forestry. Editor Bajaj Y. P. S. (Berlin, Heidelberg: Springer), Vol. 26. 10.1007/978-3-642-57970-7_15

[B190] U.S. Food and Drug Administration. 2020. FDA Approves First Treatment for COVID-19 . https://www.fda.gov/news-events/press-announcements/fda-approves-first-treatment-covid-19 [Accessed October 22, 2020].

[B191] van Dam J. E. van den Broek L. A. Boeriu C. G. (2017). Polysaccharides in Human Health Care. Nat. Prod. Commun. 12, 821–830. 10.1177/1934578X1701200604

[B192] Weinberger M. Lockshin B. (2017). When Is Cough Functional, and How Should it Be Treated? Breathe 13, 22–30. 10.1183/20734735.015216 28289448 PMC5344044

[B193] Werner A. Laccourreye O. (2011). Honey in Otorhinolaryngology: when, Why and How? Eur. Ann. Otorhinolaryngol. Head. Neck. Dis. 128, 133–137. 10.1016/j.anorl.2010.12.002 21310682

[B194] Wiersinga W. J. Rhodes A. Cheng A. C. Peacock S. J. Prescott H. C. (2020). Pathophysiology, Transmission, Diagnosis, and Treatment of Coronavirus Disease 2019 (COVID-19): A Review. JAMA 324 (8), 782–793. 10.1001/jama.2020.12839 32648899

[B195] Wise P. M. Breslin P. A. S. Dalton P. (2014). Effect of Taste Sensation on Cough Reflex Sensitivity. Lung 192, 9–13. 10.1007/s00408-013-9515-z 24173385

[B196] Yang F. Zhang Y. Tariq A. Jiang X. Ahmed Z. Zhihao Z. (2020). Food as Medicine: A Possible Preventive Measure against Coronavirus Disease (COVID-19). Phytother. Res. 34 (12), 3124–3136. 10.1002/ptr.6770 32468635 PMC7283886

[B197] Yazdi N. Kardooni M. Namjuyan F. Vardanjani H. M. Tafazoli V. Jaladat A. M. (2020). Efficacy of Sweet Violet (*Viola Odorata*) Flower Oil on the Symptoms of Adults with Allergic Rhinitis: A Double-Blind Randomized Placebo-Controlled Clinical Trial. Complement. Ther. Med. 51, 102408. 10.1016/j.ctim.2020.102408 32507426

[B198] Zahm J. M. Milliot M. Bresin A. Coraux C. Birembaut P. (2011). The Effect of Hyaluronan on Airway Mucus Transport and Airway Epithelial Barrier Integrity: Potential Application to the Cytoprotection of Airway Tissue. Matrix Biol. 30, 389–395. 10.1016/j.matbio.2011.07.003 21839834

[B199] Zaitseva O. Khudyakov A. Sergushkina M. Solomina O. Polezhaeva T. (2020). Pectins as a Universal Medicine. Fitoterapia 146, 104676. 10.1016/j.fitote.2020.104676 32561422

[B200] Zanasi A. Fontana G. Mutolo D. (2020). Cough: Pathophysiology, Diagnosis and Treatment. Gewerbestrasse: Springer International Publishing.

[B201] Zarshenas M. M. Badr P. Moein M. R. (2013). Laooq: Selective Respiratory Dosage Form Used in Medieval Persia. Pharm. Hist. (Lond). 43, 34–38. 24624709

[B202] Zhang D. H. Zhang X. Peng B. Deng S. Q. Wang Y. F. Yang L. (2020). Network Pharmacology Suggests Biochemical Rationale for Treating COVID-19 Symptoms with a Traditional Chinese Medicine. Commun. Biol. 3, 466. 10.1038/s42003-020-01190-y 32811894 PMC7434773

